# Regulation of protein kinase Cδ Nuclear Import and Apoptosis by Mechanistic Target of Rapamycin Complex-1

**DOI:** 10.1038/s41598-019-53909-5

**Published:** 2019-11-26

**Authors:** Antonio Layoun, Alexander A. Goldberg, Ayesha Baig, Mikaela Eng, Ortal Attias, Kristoff Nelson, Alexandra Carella, Nahomi Amberber, Jill A. Fielhaber, Kwang-Bo Joung, T. Martin Schmeing, Yingshan Han, Jeffrey Downey, Maziar Divangahi, Philippe P. Roux, Arnold S. Kristof

**Affiliations:** 1 Meakins-Christie Laboratories and Translational Research in Respiratory Diseases Program, Research Institute of the McGill University Health Centre, Faculty of Medicine, Departments of Medicine and Critical Care, 1001 Décarie Boulevard, EM3.2219, Montreal, Québec H4A 3J1 Canada; 2 Department of Biochemistry, McGill University, Montréal, Québec H3G 0B1 Canada; 3 Meakins-Christie Laboratories and Translational Research in Respiratory Diseases Program, Research Institute of the McGill University Health Centre, 1001 Décarie Boulevard, EM3.2219, Montréal, Québec H4A 3J1 Canada; 4 Institute for Research in Immunology and Cancer, Faculty of Medicine, University of Montreal, P.O. Box 6128, Station Centre-Ville, Montréal, Québec H3C 2J7 Canada

**Keywords:** Cell death, Cell signalling, Nuclear transport

## Abstract

Inactivation of the protein complex ‘mechanistic target of rapamycin complex 1’ (mTORC1) can increase the nuclear content of transcriptional regulators of metabolism and apoptosis. Previous studies established that nuclear import of signal transducer and activator of transcription-1 (STAT1) requires the mTORC1-associated adaptor karyopherin-α1 (KPNA1) when mTORC1 activity is reduced. However, the role of other mTORC1-interacting proteins in the complex, including ‘protein kinase C delta’ (PKCδ), have not been well characterized. In this study, we demonstrate that PKCδ, a STAT1 kinase, contains a functional ‘target of rapamycin signaling’ (TOS) motif that directs its interaction with mTORC1. Depletion of KPNA1 by RNAi prevented the nuclear import of PKCδ in cells exposed to the mTORC1 inhibitor rapamycin or amino acid restriction. Mutation of the TOS motif in PKCδ led to its loss of regulation by mTORC1 or karyopherin-α1, resulting in increased constitutive nuclear content. In cells expressing wild-type PKCδ, STAT1 activity and apoptosis were increased by rapamycin or interferon-β. Those expressing the PKCδ TOS mutant exhibited increased STAT1 activity and apoptosis; further enhancement by rapamycin or interferon-β, however, was lost. Therefore, the TOS motif in PKCδ is a novel structural mechanism by which mTORC1 prevents PKCδ and STAT1 nuclear import, and apoptosis.

## Introduction

‘Mechanistic target of rapamycin’ (mTOR) is a conserved kinase that integrates anabolic or cell stress signals to control transcriptional, translational, and post-translational programs which regulate metabolism, growth, and survival^1,2^. mTOR coordinates two distinct macromolecular protein complexes; mTOR complex 1 (mTORC1) phosphorylates signaling intermediates (*e.g*., S6K, 4E-BP1) that control protein synthesis, cell growth, and cell proliferation. mTORC1 activity is blocked by rapamycin, a drug used for transplant immunosuppression or cancer chemotherapy, and contains the protein adaptor raptor^3–5^. Its effector proteins can be physically coupled to mTORC1 in a mechanism that requires their ‘target of rapamycin signaling’ (TOS) motifs^6^. mTORC2 contains the adaptor protein rictor, exhibits resistance to the acute effects of rapamycin, and regulates cytokinesis and cell survival via distinct effector kinases^7^.

Emerging studies indicate that mTORC1 drives cell metabolism or survival in part by regulating the nuclear content of transcriptional regulators^2,8,9^. For instance, when inactivated (*e.g*., by rapamycin or nutrient restriction), mTORC1 coordinates the nuclear import of ‘signal transducer and activator of transcription 1’ (STAT1), thereby controlling the induction of apoptosis or autophagy genes^10,11^. This requires their physical association with the nuclear import adaptor karyopherin-α1 (KPNA1), as well as the mTORC1-associated phosphatase subunits α4 and PP2Ac^12,13^. Importantly, blockade of mTORC1 with rapamycin increased the nuclear content of *latent* (*i.e*., unphosphorylated) STAT1, which modifies distinct transcriptional responses related to apoptosis and autophagy; this occurs independently of STAT1 phosphorylation at the residues required for transcriptional activation (*i.e*., S727) or interferon-activated nuclear import (*i.e*., Y701)^10,14–16^. Although the regulation of nuclear import by mTORC1 via de-phosphorylation is conserved^12,17^, and nuclear import is regulated by phosphorylation, the complement of kinases and phosphatases, and their target residues in karyopherins, coordinating mTORC1-dependent nuclear import remains poorly understood^18^.

**Figure 1:**
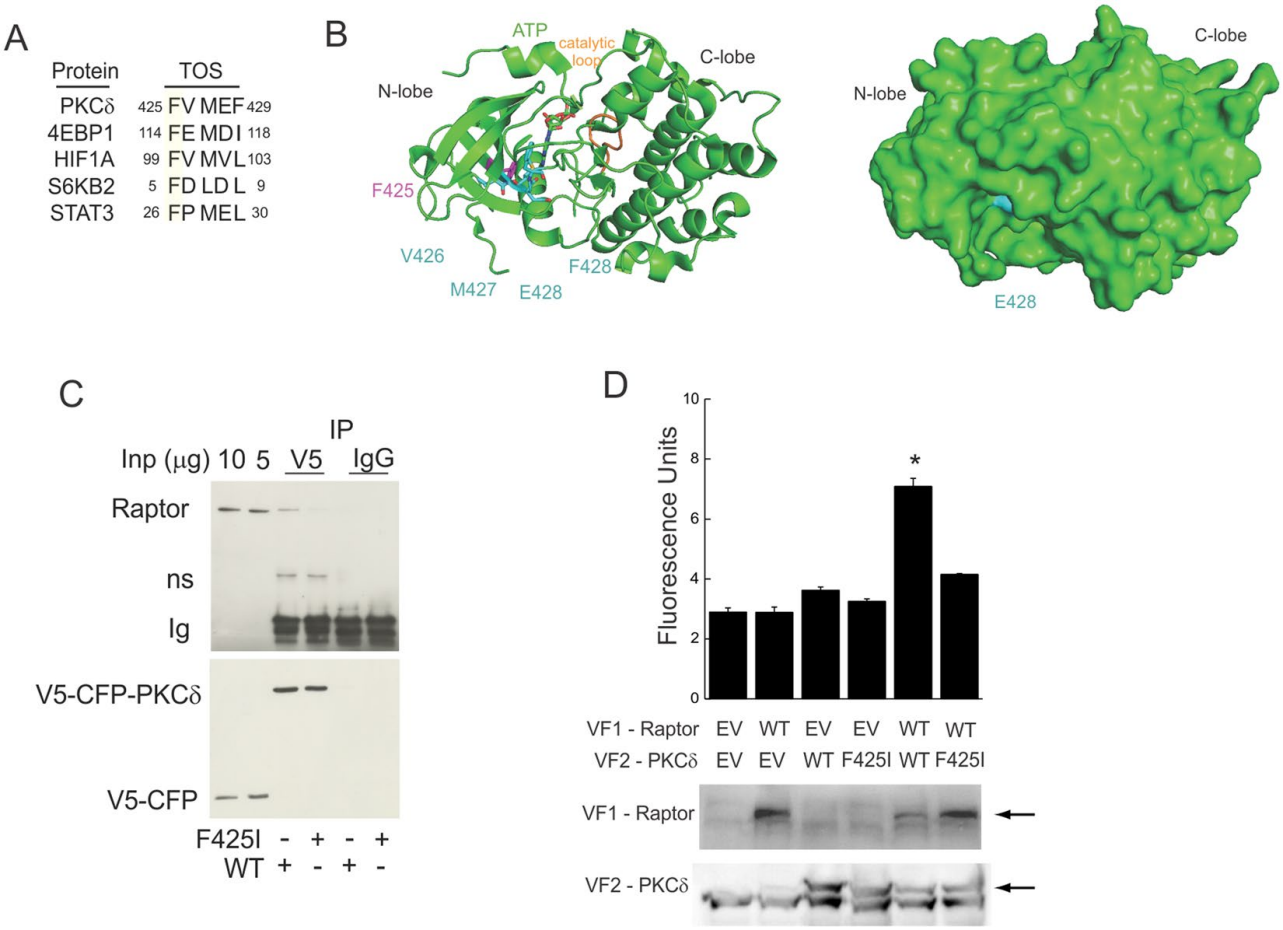
A TOS motif in PKCδ drives its physical interaction with raptor in intact cells. (**A**) Sequence alignment indicating the primary sequence of TOS motifs in mTORC1-interacting proteins. (**B**) Structure of the kinase domain of protein kinase B (REF DOI: 10.1038/nsb870) is homologous to that in PKCδ. Cartoon representation with the TOS motif and ADPCP in sticks (left). Surface representation is shown on the right. E428 was changed from D and F428 from Y to visualize the proper sequence of the TOS motif. The images were prepared using the program Pymol 2.0. (**C**) After expression of V5-CFP-WT PKCδ or V5-CFP-PKCδ F425I in HEK293T cells, raptor or V5-containing recombinant proteins were detected by Western blot in complexes affinity purified using αV5 antibody. (**D**) COS7 cells were transfected with plasmids for the mammalian expression of complementary fragments of yellow fluorescence protein (VF1, VF2) alone (EV) or those linked to recombinant PKCδ (WT, F425I) or raptor (WT) before detection of fluorescence in cell lysates (Top) or proteins by Western blot (Bottom). Data represent 3–5 experiments. Fluorescence assays were performed using duplicate samples.

As a first step, we had identified the pro-apoptotic kinase ‘protein kinase C-delta’ (PKCδ) as a component of the mTORC1/STAT1 complex^19^, which on sequence analysis contains a TOS motif (Fig. 1). The pro-apoptotic kinase PKCδ was previously identified in endogenous mTOR-containing complexes, contained an mTORC1-sensitive phosphorylation site, and could phosphorylate STAT1^20–22^. The nuclear trafficking of PKCδ was required for the induction of apoptosis in a caspase-3-dependent mechanism^23^. We hypothesized that mTORC1 activity suppresses PKCδ nuclear content and apoptosis via a physical interaction that requires its intact TOS motif. Conversely, a loss of function mutation in the PKCδ TOS motif (F425I) should disrupt its interaction with mTORC1, and disable mTORC1-dependent suppression of its nuclear import. Here, expanding on previous and current work demonstrating PKCδ in a complex with mTORC1, and not mTORC2, we show that the putative TOS motif in PKCδ directs its association with mTORC1. Moreover, mutation of TOS motif uncouples PKCδ from its regulation by mTORC1 or KPNA1, enhances the constitutive nuclear content of PKCδ, and increased rapamycin-induced apoptosis.

## Results

### PKCδ associates with raptor in intact cells

We previously showed that endogenous mTOR, PKCδ, and STAT1 are detected in a macromolecular complex that undergoes nuclear trafficking^13,19^. The STAT1/PKCδ heterodimer translocated to the nucleus in cells exposed to rapamycin, suggesting co-regulation by, or physical association with, mTORC1. *In silico* analysis revealed a consensus ‘target of rapamycin signaling’ (TOS) motif in human PKCδ between amino acid residues 425 and 429 (N′- FVMEF – C′); the amino acid sequence resembled that found in other known mTORC1-bound effector proteins that associate with raptor (Fig. 1A). Structural analyses predicted the TOS motif to reside within a highly packed hydrophobic region of PKC, and not at the protein surface, with F425 (Fig. 1A) facing the interior of the molecule (Fig. 1B - pink). In previous studies, mutation of the N-terminal phenylalanine to a less bulky non-polar amino acid (*i.e*., isoleucine, alanine) prevented the interaction between raptor and TOS-containing proteins^24–26^. In cells expressing recombinant V5-PKCδ, raptor was associated with the wild-type isoform, but not that containing a mutated TOS sequence (phenylalanine 425 to isoleucine; F425I) (Fig. 1C). By protein fragment complementation assay (PCA), a physical interaction was detected between raptor and wild-type PKCδ, but not with the F425I mutant (Fig. 1D). Whereas the PKCδ F425I mutant was stable and expressed in the cytoplasm, that with a F425 to alanine mutation (F425A) appeared trapped in the Golgi apparatus when expressed in COS7 cells, and was not detected by Western blot (Supplementary Fig. S1A,B). In control assays, PKCδ co-eluted with mTOR and raptor, but not rictor, in ion exchange chromatography fractions previously shown^13^ to contain STAT1 and the mTOR-associated phosphatases (Supplementary Fig. S1D). In agreement, PKCδ was associated with recombinant or endogenous raptor (Supplementary Fig. S1E,F). Therefore, consistent with previous work^19,20^, endogenous or recombinant PKCδ can be physically associated with mTORC1. Their association in live cells requires an intact TOS motif. Mutation of the TOS motif F425 to progressively less bulky hydrophobic moieties likely modifies the folding properties of PKCδ, and leads to loss of interaction with mTORC1, abnormal trafficking, and/or instability.

### Physical association between PKCδ and the nuclear import adaptor KPNA1

**Figure 2:**
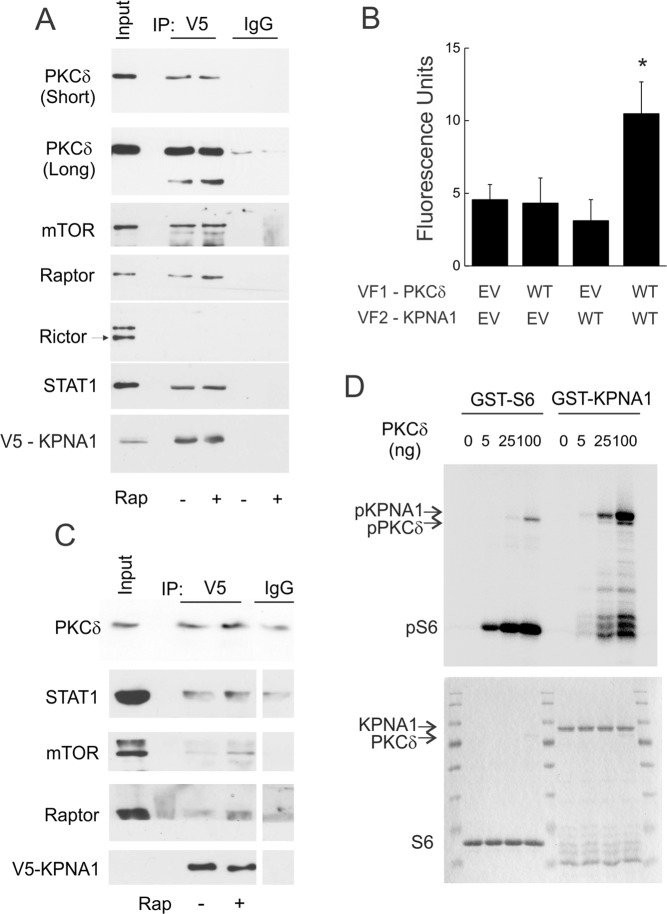
PKCδ physically interacts with the nuclear import adaptor KPNA1. (**A**) HEK293T cells expressing V5-CFP-KPNA1 were incubated without or with rapamycin, 50 ng/ml, for 30 min. The indicated KPNA1-interacting proteins were detected by Western blot, after immunoprecipitation with αV5 antibody. (**B**) COS7 cells were transfected with plasmids for the mammalian expression of complementary fragments of yellow fluorescence protein (VF1, VF2) alone (EV) or those linked to recombinant PKCδ (WT) or KPNA1 (WT) before detection of fluorescence in cell lysates. *p < 0.05 *vs*. empty vector control (lane 1). (**C**) HEK293T cells expressing V5-CFP-KPNA1 were exposed to rapamycin, 50 ng/ml, for 0 or 30 min before preparation of nuclear lysates. The indicated proteins were detected in V5 immunoprecipitates. Composite images from the same gels are shown, with lanes separated by a vertical white line. (**D**). Recombinant PKCδ, 0–100 ng, was incubated with purified GST-S6 or GST-KPNA1 in the presence of [γ^32^P]-ATP in an *in vitro* kinase reaction. Products were separated by SDS-PAGE before autoradiography (top) or staining with Coomassie blue (bottom). Data are representative of 3–6 individual experiments.

We previously demonstrated that endogenous KPNA1, PKCδ, or STAT1 are detected in mTOR immunoprecipitates^12,19^, suggesting a functional association between KPNA1 and PKCδ. To confirm that recombinant KPNA1 can associate with endogenous mTORC1 and PKCδ, we detected proteins from V5 immunoprecipitates in cells expressing V5-CFP-KPNA1 (Fig. 2A); these included mTOR, PKCδ, STAT1, and raptor but not rictor. In the same experiment, phosphorylation of p70 S6 kinase (S6K) was blocked by rapamycin, indicating inhibition of mTORC1 activity (Fig. S1G). Furthermore, by protein fragment complementation assay, recombinant PKCδ and KPNA1, each linked to a complementary fragment of yellow fluorescence protein (*i.e*., VF1, VF2), interacted with each other, but not with VF1 or VF2 alone (Fig. 2B). The mTORC1 inhibitor rapamycin did not modify the association between PKCδ and recombinant KPNA1 in whole cell extracts (Fig. 2A), but led to the nuclear enrichment of complexes containing KPNA1, PKCδ, STAT1, raptor, and mTOR when compared to non-specific binging to the sepharose beads (Fig. 2C; IP V5 + rap *vs*. IgG). In the same experiment, analysis of nuclear, cytosolic, and membrane fractions by Western blot indicated nuclear enrichment of mTOR, raptor, PKCδ, endogenous KPNA1, and recombinant KPNA1 in cells exposed to rapamycin (Fig. S2); rictor (mTORC2) was primary expressed in the membrane fraction, and, unlike raptor, did not undergo nuclear enrichment in response to rapamycin. By *in vitro* kinase assay (Fig. 2D), recombinant KPNA1 (upper band) was phosphorylated by purified recombinant PKCδ (lower band) in concentration-dependent fashion. These data indicate that KPNA1 can associate with endogenous PKCδ and mTORC1 in a complex that becomes more abundant in the nucleus when cells are exposed to rapamycin. Moreover, gel purification of endogenous protein complexes (Fig. S1D), affinity purification with recombinant KPNA1 (Fig. 2A), or subcellular fractionation (Fig. S2), indicate a nuclear trafficking complex containing mTORC1 (raptor), but not mTORC2 (rictor). These results suggest that the TOS motif in PKCδ might be required for mTORC1- and KPNA1-dependent regulation of PKCδ nuclear import.

### KPNA1 is required for the nuclear import of endogenous PKCδ under conditions of reduced mTORC1 activity

**Figure 3:**
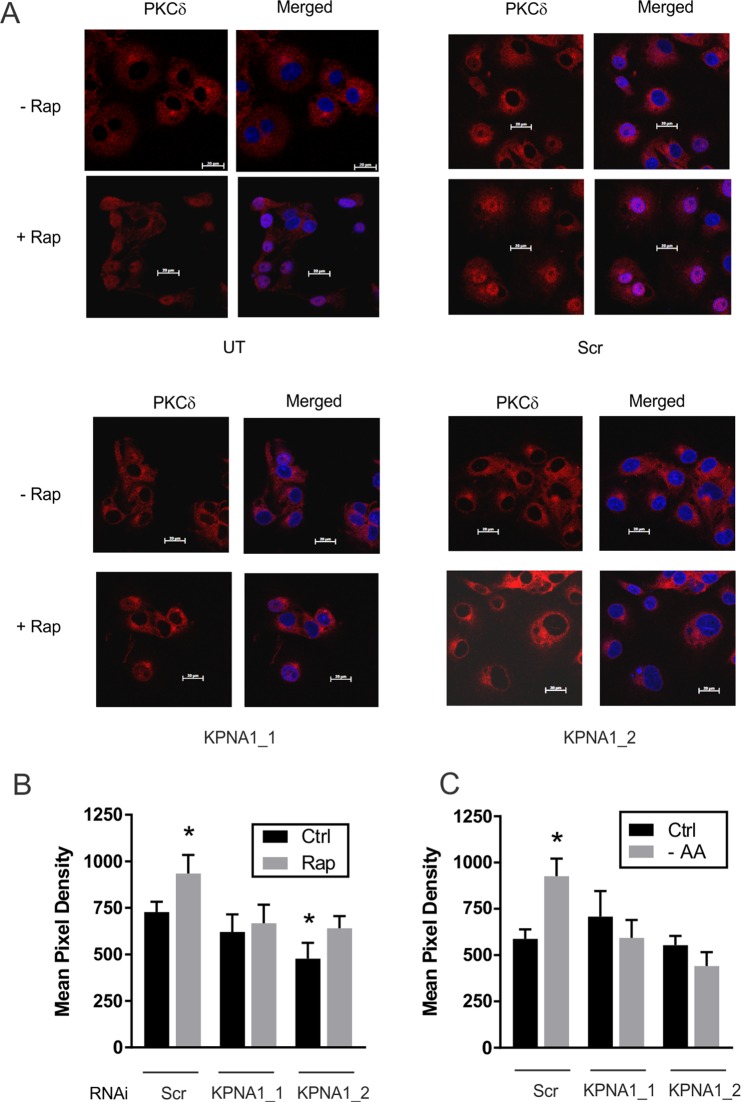
mTORC1 and KPNA1 regulate endogenous PKCδ nuclear content. (**A**) COS7 cells expressing control scrambled (Scr) shRNAs, or those expressing shRNAs targeting two distinct sequences in KPNA1 (KPNA1_1, KPNA1_2) were exposed to vehicle (-Rap), rapamycin (+Rap), 50 ng/ml, or media lacking leucine, arginine, and lysine (−AA) for 1 h before immunofluorescence confocal imaging of PKCδ. Representative images were acquired using a 63x objective. Mean nuclear pixel densities (n = 7–8 fields with a 20X objective ± SEM) for cells exposed to (**B**) rapamycin or (**C**) amino acid restriction (−AA) are shown. *p < 0.05 *vs*. control in cells expressing control shRNA.

Previous studies indicated regulation of PKCδ nuclear import by mTORC1^13^. We next determined whether KPNA1 is required. COS7 cells were transduced with lentiviruses for the expression of non-targeting short hairpin RNAs (shRNAs) or those targeting distinct coding sequences in KPNA1 (*i.e*., KPNA1_1, KPNA1_2; Fig. 3); specificity was confirmed previously for KPNA1_1^12^, or by re-assessing the expression of genes or proteins other than KPNA1 in cells expressing KPNA1_1 or KPNA1_2 shRNAs (Supplementary Fig. S3A,B). In untransduced cells (UT), or those expressing a non-targeting shRNA control (Scr), the mTORC1 inhibitor rapamycin led to a significant increase in PKCδ nuclear import when compared to vehicle control (Fig. 3A, top panels). Expression of two distinct shRNAs targeting KPNA1 (KPNA1_1, KPNA1_2) blocked the rapamycin-induced nuclear import of endogenous PKCδ (Fig. 3A, bottom panels); effects of KPNA1 depletion were quantified by image analysis (Fig. 3B). The same effects of KPNA1 depletion were observed for amino acid restriction, a known physiological inhibitor of mTORC1 activity (Fig. 3C, S3C).

### The TOS motif is required for the attenuation of PKCδ nuclear import by mTORC1

**Figure 4:**
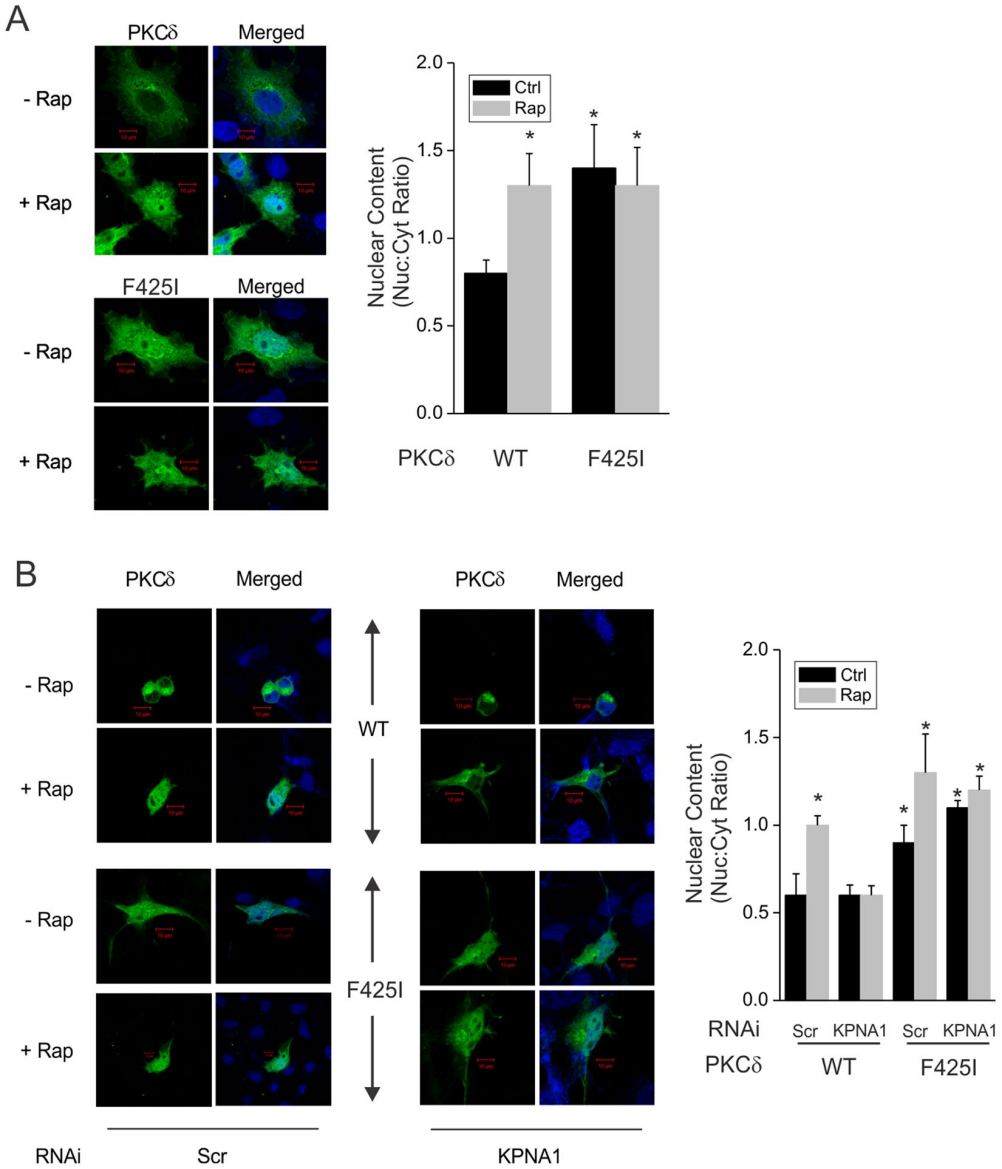
The TOS motif in PKCδ and KPNA1 mediate the effect of mTORC1 on PKCδ nuclear content. (**A**) COS7 cells expressing GFP-tagged wild-type PKCδ (GFP-WT-PKCδ) or TOS mutated PKCδ (GFP-PKCδ F425I) were exposed to vehicle or rapamycin, 50 ng/ml, for 1 h before fixation and confocal imaging. In (**B**), GFP-PKCδ-expressing COS7 cells were co-infected with lentiviral constructs for expression of αKPNA1 shRNA or scrambled control (Scr). Shown to the right of images are the means of PKCδ nuclear:cytoplasmic ratio ± SEM (n = 5–8 cells). *p < 0.05 *vs*. vehicle WT-PKCδ-expressing control.

To better define the mechanism by which mTORC1 controls PKCδ nuclear import, green fluorescent protein (GFP)-tagged recombinant wild-type (WT) or mutant TOS (F425I) PKCδ were expressed in COS7 cells. We used rapamycin to elicit inactivation of mTORC1. As was the case for endogenous PKCδ, rapamycin triggered the nuclear import of WT-PKCδ (Fig. 4A – upper panel). In contrast to the WT form, the TOS-mutant exhibited increased constitutive nuclear localization (Fig. 4A – lower panel), indicating that the TOS motif favours retention of PKCδ by mTORC1 in the cytoplasm. Depletion of KPNA1 by RNAi prevented the nuclear translocation of WT-PKCδ in cells exposed to rapamycin (Fig. 4B – upper right panel); depletion of KPNA1 in cells expressing the TOS mutant of PKCδ failed to reduce its nuclear content (Fig. 4B – lower right panel), likely due to its uncoupling from mTORC1 and KPNA1. Therefore, the TOS motif in PKCδ permits its enhanced nuclear import under conditions of reduced mTORC1 activity, via a mechanism that requires the mTORC1-associated nuclear import adapter KPNA1. In the absence of a functional TOS motif, PKCδ undergoes nuclear import, but is no longer regulated by mTORC1 or KPNA1.

### Augmentation of STAT1 nuclear import and activity by rapamycin is blocked by mutation of the PKCδ TOS motif

**Figure 5:**
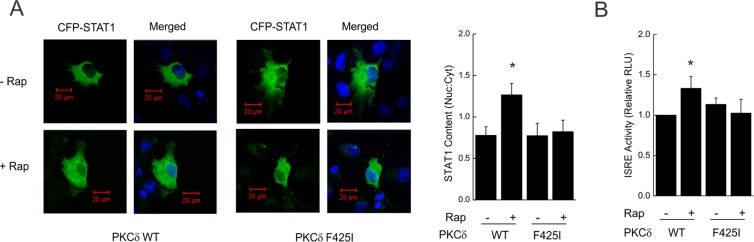
The PKCδ TOS motif is required for the enhancing effect of rapamycin on STAT1 nuclear content and activity. (**A**). COS7 cells were co-transfected with plasmids for the expression of V5-tagged PKCδ isoforms (WT or F425I) or CFP-V5-STAT1 before exposure to vehicle (-Rap) or rapamycin (+Rap), 50 ng/ml, for 60 min and confocal imaging. Values for mean nuclear:cytoplasmic ratio (n = 3–5 cells per condition) ± SEM are shown to the right. (**B**) A549 cells were transfected with plasmids containing a luciferase reporter construct driven by interferon stimulated response elements (ISRE) before exposure to vehicle or rapamycin for 6 h, and assessment of luciferase activity in cell lysates. Data are the means of normalized luciferase activity from 3 experiments ± SEM, each performed in triplicate. *p < 0.05 vs. control.

In previous studies, inactivation of mTORC1 with rapamycin increased endogenous STAT1 nuclear content independent of its canonical phosphorylation at Y701 or S727^12,13^. We reasoned that the PKCδ TOS motif might play a role in the nuclear import of latent (*i.e*., unphosphorylated) STAT1. Recombinant WT PKCδ and CFP-V5-STAT1 were co-expressed in COS7 cells. Although total levels of CFP-V5-STAT1 may have varied, rapamycin enhanced the nuclear content of STAT1 (nuclear:cytoplastic ratio). The effect of rapamycin was lost in cells expressing the TOS mutant (Fig. 5A). In similar fashion, rapamycin increased STAT1 activity (*i.e*., luciferase activity driven by the interferon-stimulated response element (ISRE, Fig. 5B) in cells expressing WT PKCδ, but not in those expressing the TOS mutant. Rapamycin had no effect on the activity of a reporter for IFN-γ-activated STAT1 homodimers (GAS, Supplementary Fig. S4A). Phosphorylation of STAT1 at the known PKCδ-sensitive residue (*i.e*., S727) was blocked by expression of a kinase-dead PKCδ mutant, but not by the TOS mutant, indicating that the latter retains kinase activity (Supplementary Fig. S4B). Moreover, ISRE activation by IFN-γ or IFN-β was retained in cells expressing the TOS mutant (Fig. S4C).

### The TOS motif is required for mTORC1 suppression of programmed cell death

**Figure 6:**
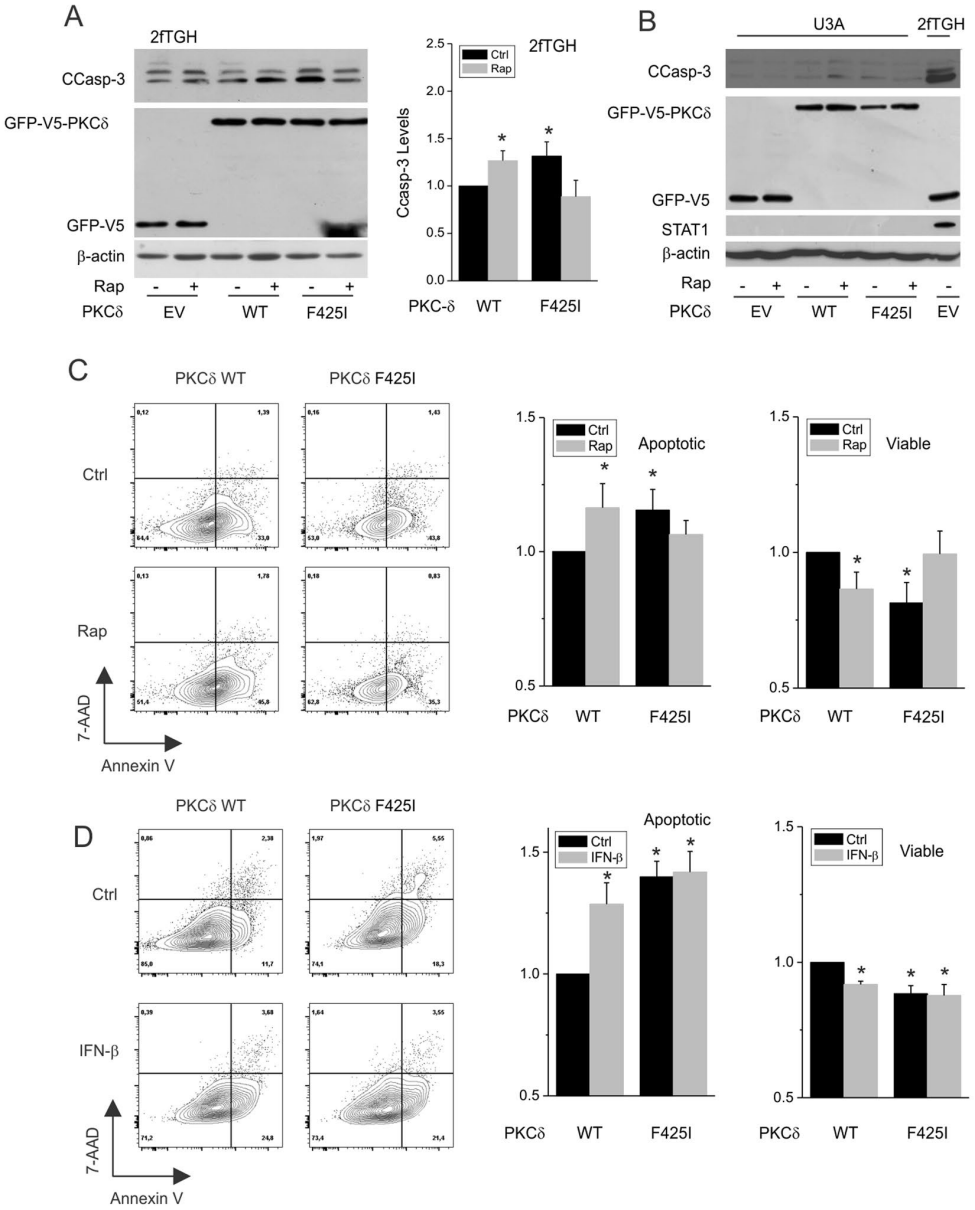
Role of mTORC1 in the regulation of apoptosis by PKCδ. (**A**) 2fTGH cells or (**B**) their STAT1-deficient counterparts (U3A) were exposed to vehicle or rapamycin, 50 ng/ml, for 24 h before detecting levels of cleaved caspase-3 (CCasp-3) by Western blot. For A., summarized data are shown to the right of a representative gel, and represent the means of cleaved caspase-3 levels ± SEM from 8 individual experiments. In (**C**,**D**) GFP-PKCδ-expressing COS7 cells were exposed to rapamycin, 50 ng/ml (**C**) or IFN-β, 250 U/ml (**D**) for 24 h, before evaluation by flow cytometry for Annexin V binding and staining with 7-AAD. The proportion of Annexin V- and GFP-positive (apoptotic) cells are shown, as well as the number of viable cells. Data (**A**,**C**,**D**) are the means of fold changes in cleaved caspase-3 (Ccasp3), apoptosis, or viability, respectively, ± SEM from 3–5 individual experiments each performed in duplicate. *p < 0.05 vs. control.

Apoptosis was evaluated as a biological consequence of early latent STAT1 nuclear import and transcriptional activity. We previously used human sarcoma (2fTGH) cells and their STAT1-deficient counterparts (U3A) to investigate mTORC1-regulated STAT1 activity and apoptosis^10,12^. Like ISRE activity and STAT1 protein, levels of cleaved caspase-3 were increased in human sarcoma (2fTGH) cells expressing the F425I TOS mutant (Fig. 6A - lane 5), and the enhancing effect of rapamycin on apoptosis was lost (Fig. 6A – lane 6). Expression of the PKCδ TOS mutant in STAT1-deficient U3A cells failed to restore apoptosis (Fig. 6B). Thus, although mTORC1 and PKCδ can modulate rapamycin-induced apoptosis, a STAT1-dependent transcriptional program^27^ is required.

**Figure 7:**
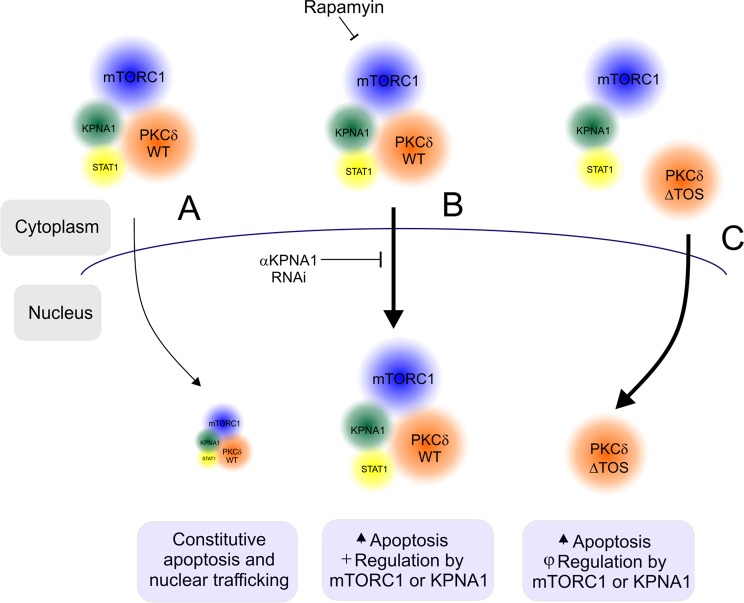
Summary: (**A**) The complex containing mTORC1 (blue), PKCδ (orange), STAT1 (yellow), and KPNA1 (green) undergoes constitutive nuclear trafficking that is (**B**) enhanced by inactivation of mTORC1 (rapamycin), and blocked by depletion of KPNA1. (**C**) Disruption of the interaction between PKCδ and mTORC1 by mutation of the TOS motif in PKCδ (ΔTOS; F425I) leads to enhanced constitutive nuclear content of PKCδ, and loss of regulation by mTORC1 or KPNA1. Apoptosis is enhanced by rapamycin in cells expressing wild-type PKCδ, but not in those expressing the mTORC1 interaction mutant (ΔTOS).

To evaluate cell autonomous effects of PKCδ on apoptosis in a second cell line, COS7 cells were transfected to express the recombinant WT or GFP-PKCδ F425I, and apoptosis was assessed by flow cytometry (Fig. 6C,D). Apoptosis in GFP-WT-PKCδ-expressing cells was increased by rapamycin. As was the case for PKCδ nuclear content and STAT1 activity, cells expressing the TOS mutant exhibited increased basal apoptosis, and further enhancement by rapamycin (Fig. 6C) or IFN-β (Fig. 6D) was lost; observed increases in apoptosis were consistent with previous studies^28–30^, and associated with corresponding reductions in cell viability (Fig. 6C,D). Taken together, the results indicate a physical interaction between mTORC1 and PKCδ that requires a conserved TOS motif in PKCδ (summarized in Fig. 7). The mTORC1- regulated alpha karyopherin KPNA1 can also interact with PKCδ; KPNA1 is required for PKCδ nuclear import under conditions of reduced mTORC1 activity. Uncoupling of PKCδ from the inhibitory effect of mTORC1 (*i.e*., by mutation of the PKCδ TOS motif) augments PKCδ nuclear content, STAT1 activity, and apoptosis, and prevents a further enhancing effect of rapamycin.

## Discussion

The current study reveals a novel ‘target of rapamycin signaling’ (TOS) structural motif in PKCδ required for the suppressive effect of mTORC1 on PKCδ nuclear import and apoptosis. The inactivation of mTORC1 by rapamycin or nutritional restriction leads to a highly-conserved stress response involving the attenuation of energy-requiring processes (*e.g*., protein synthesis, cell growth and proliferation, ribosomal biogenesis), and the initiation of adaptive responses (*e.g*., autophagy, utilization of alternative energy sources)^31,32^. One important but poorly understood adaptive mechanism during mTORC1 blockade is the enhanced nuclear content of transcription factors that control the expression of genes for metabolic adaptation or apoptosis (*e.g*., TFEB, STAT1)^13,33,34^. Here, we show that the TOS motif in PKCδ facilitates its physical interaction with raptor (*i.e*., mTORC1). KPNA1, an mTORC1-regulated cargo-specific nuclear import adaptor for STAT1, also interacted with PKCδ, and was required for the nuclear import of endogenous or recombinant PKCδ upon inactivation of mTORC1 (*i.e*., rapamycin, amino acid restriction). Rapamycin failed to further augment PKCδ nuclear content, STAT1 activity or apoptosis in cells expressing a form of PKCδ that cannot associate with mTORC1 (PKCδ F425I). The PKCδ TOS mutant exhibited constitutively increased nuclear content, which correlated with elevated basal STAT1 activity and apoptosis. Therefore, a newly recognized TOS motif in PKCδ is required for the regulation of constitutive PKCδ and STAT1 nuclear trafficking and apoptosis by mTORC1.

The highly-conserved TOS structural motif (residues FXϕD/Eϕ, where X is any amino acid and ϕ is hydrophobic) specifies selected mTORC1-interacting proteins^35,36^. For 4E-BP1 and p70 S6 kinase, the TOS motif coordinates their interaction with the FRB domain of mTOR via direct binding to raptor^37,38^. For PKCδ, TOS motif residues 425–428 form the last half of the final β-strand in the PKCδ kinase domain N-lobe, and residue 429 starts the loop that bridges to the C-lobe (Fig. 1B). Although they do not make any side-chain specific hydrogen bonds to ATP, residues 427–429 form part of the adenosine binding pocket. Apart from the side chain of E428, the TOS motif is essentially inaccessible for direct binding of partner proteins. Moreover, the activity or phosphorylation of STAT1 at S727 in cells exposed to interferons was retained (Supplementary Fig. S3), suggesting that the TOS mutant of PKCδ interferes with its interaction with mTORC1 independent of PKCδ kinase activity. Future studies might identify other PKC isoforms that interact with mTORC1 by interrogating their sequences for TOS motifs. Both PKCδ and PKCƐ contain mTORC1-sensitive phosphorylation sites^22^, and can mediate IFN-induced cellular responses^21^, but little is known regarding the regarding the regulation of other PKC isoforms by mTOR.

The nuclear content of the PKCδ TOS mutant was not altered by inhibition of mTORC1 or depletion of KPNA1, suggesting an alternative mechanism for PKCδ nuclear trafficking (Fig. 4B). These might include PKCδ interaction with another α-karyopherin, or its nuclear import by β-karyopherin in the absence of an α-karyopherin^39^. Regarding the former possibility, PKCδ was associated with KPNA2 during its nuclear import in response to H_2_O_2_ or etoposide^40^. In this case, the nuclear content of PKCδ was controlled by post-translational modification of its nuclear localization sequence. Therefore, there are at least two mechanisms for regulation of PKCδ nuclear import and pro-apoptotic activity, one of which requires its interaction with mTORC1 which is mediated by a conserved TOS motif.

A functional association between PKCδ, mTORC1, and KPNA1, as well as control of KPNA1 by mTORC1, is supported by current (Figs. 1, 2, S1) and previous^12,13^ biochemical studies. In keeping, the mTORC1-associated phosphatase PP2Ac regulates the phosphorylation of recombinant KPNA1^12^, and PKCδ exhibits kinase activity for KPNA1 *in vitro* (Fig. 2D). We and others have shown that the interaction between mTORC1 and PKCδ does not require mTOR kinase activity *per se*^13,19,41^. Ongoing studies on post-translational modification of KPNA1 by mTORC1 effectors, or how mutation of the TOS motif affects other interactions within the complex, will better delineate the molecular mechanism(s) by which mTORC1 controls the phosphorylation of KPNA1 and the nuclear import of its cargo in intact cells. These include the identification of mTOR-, PKCδ-, and PP2Ac-sensitive target residues in KPNA1, and the use of their kinase- or phosphatase-dead isoforms to better understand the biochemical events governing the regulation of nuclear import by mTORC1. This paradigm is evolutionarily conserved, as the yeast homologues of α4 and PP2Ac (*i.e*., Tap42, Pph, Sit4) also mediate TOR-dependent suppression of nuclear import via the yeast karyopherin-α homologue Srp1^42,43^, and Srp1 is a substrate for phosphorylation *in vitro* and in intact cells^44,45^.

The current study demonstrates that rapamycin increases the nuclear content of PKCδ and STAT1 under conditions which we have previously shown to be independent of STAT1 phosphorylation^13^. In contrast other studies on IFN-stimulated cells established PKCδ as a kinase for STAT1 at S727, thereby promoting STAT1 transcriptional activity. Interferons induce the canonical phosphorylation of STAT1 at Y701, dimerization, nuclear translocation, binding to GAS enhancer sites in target genes (Supplementary Fig. S3), and the expression of genes involved in innate immunity^14,27,46,47^. Our previous results indicated that mTORC1 inhibition with rapamycin does not modify phosphorylation of STAT1 at S727 or Y701^13,19^. Other studies support an emerging role for latent (*i.e*., unphosphorylated) STAT1 as a regulator of distinct transcriptional responses. That is, STAT1, or its Y701F (unphosphorylated) mutant, constitutively occupied the ‘low molecular mass peptide 2’ (*LMP2*) gene promoter, and supported the transcription of *LMP2*, a gene involved in immune modulation^16^. Stable expression of the STAT1 Y701F mutant, a model for latent STAT1, prolonged the expression of a subset of interferon-sensitive genes involved in anti-viral responses and immune regulation, including *STAT1* itself^48^. The current study complements previous work on the control of latent STAT1 activity by mTORC1^11–13^, and establishes PKCδ as a regulator of mTORC1-dependent nuclear trafficking and cell survival.

Rapamycin blocked apoptosis in cells expressing the PKCδ F425I mutant (Fig. 6), perhaps by unmasking pro-apoptotic functions of mTORC1. Other have postulated that the effect of mTORC1 on cell death or survival may reflect the balance between its pro- and anti-apoptotic effects^49^. Several studies demonstrated that mTORC1 was required for ER-stress- or interferon-α-induced apoptosis in mechanisms that are independent of *de novo* gene transcription^50,51^. Our results suggest that changes in the composition (*e.g*., loss of PKCδ) or localization (*e.g*., nuclear vs. cytoplasmic) of mTORC1 complexes may be an important determinant of death or survival.

mTORC1 inhibitors are currently approved for the treatment of selected cancers, with the intent of suppressing cell growth and proliferation. However, inhibition of mTORC1 also blocks a negative feedback mechanism to mitogenic PI3K/Akt signaling that paradoxically results in enhanced cell survival. This limits the therapeutic efficacy of mTORC1 inhibitors to suppression of tumor growth in inherited tumor suppressor syndromes related to excessive mTORC1 activity (*e.g*., Tuberous Sclerosis Complex and Lymphangioleiomyomatosis, Proteus Syndrome, Cowden’s disease). Molecular mechanisms downstream of mTORC1 that mediate its attenuation of apoptosis are therefore attractive therapeutic targets for achieving tumor regression. In a proof of principle, we show that blocking the inhibitory effect of mTORC1 on PKCδ nuclear trafficking promotes apoptosis in transformed cell lines.

## Methods

### Cell culture and transfection

Human embryonic kidney (HEK) 293 T, human alveolar cell carcinoma (A549), and parental (2fTGH) or STAT1-deficient (U3A) human fibrosarcoma cells were cultured as previously described^19,52^. COS7 cells were cultured in DMEM, complete RPMI, or RPMI lacking leucine, arginine, and lysine each supplemented with 10% fetal bovine serum, penicillin, 100 U/ml, and streptomycin, 100 µg/ml. Cells were incubated without or with or rapamycin (EMD), 50 ng/ml, for the indicated times. For the heterologous expression of recombinant proteins or the transfection of promoter-driven luciferase constructs, sub-confluent cells were incubated with serum free medium, 0.5–1.0 μg plasmid DNA per 9.6 cm^2^ culture surface area, and lipofectamine 2000 or LTX (Invitrogen)^13^. HEK293T cells were transfected using calcium phosphate/DNA precipitates for 5 h or 24 h in complete medium, and incubated with Dulbecco’s modified Eagle medium containing 0.5% bovine serum albumin for 16 to 18 h prior to experimental interventions. For KPNA1 depletion by RNAi, we used the lentiviral vector pLKO.1 containing DNA encoding short hairpin RNAs (KPNA1_1 or KPNA1_2) targeting distinct sequences in the KPNA1 coding region or in no known human gene (scrambled negative control) (MISSION shRNAs TRCN0000065302 and TRCN0000065301, Sigma). Plasmids were introduced into HEK293T with lentiviral packaging vectors pRSV-REV, pMDLg/pRRE and pCMV-VSV-G using calcium chloride. Viruses were collected 72 h later, and cells co-expressing GFP-PKCδ isoforms were infected with the collected viruses in the presence of polybrene for 3 d before assessing the cellular localization of PKCδ by confocal imaging.

### Plasmids for expression of recombinant proteins

The cDNAs encoding raptor, or wild-type PKCδ were cloned by PCR into the Gateway pDONR 221 Entry vector (Invitrogen) using the plasmid pcDNA3-PKCδ (kind gift of Dr. S. Listwak, National Institutes of Health) or pRK5-HA-Raptor (from Dr. D. Sabatini via Addgene^4^) as template with oligonucleotide primers listed in Supplementary Table S1, and verified by automated sequencing. Mutations in TOS motif or active site of the pDONR 221 PKCδ template were made by site-directed mutagenesis (kit) using primers indicated in Supplementary Table S2. The wild-type or mutant PKCδ or raptor cDNAs were transferred to the gateway destination vectors pcDNA3.1/nV5/GFP-DEST, pcDNA3.1/VF1/DEST or pcDNA3.1/VF2/DEST as previously described^13^. Construction of the plasmids for mammalian expression of CFP-STAT1 or KPNA1, or bacterial expression of GST-KPNA1 was previously described^12,13^.

### Preparation of whole cell or nuclear lysates for detection of proteins or protein complexes

Endogenous or recombinant proteins in whole cell or fractionated lysates were detected by immunoprecipitation or Western blot analysis using antibodies listed in Supplementary Table S3 (methodology described as in reference 12). Whole cell lysates were generated after washing cells once with cold PBS, and incubating for 15 min on ice in lysis buffer (20 mM Tris pH 8.0, 0.3% CHAPS, 1 mM EDTA, 10 mM β-glycerophosphate, aprotinin, 10 μg/ml, leupeptin, 10 μg/ml, 1 mM PMSF, 50 mM NaF, 100 μM sodium orthovanadate). After freezing and thawing, cells were homogenized and centrifuged at 1000 × g for 5 min. Supernatants were then centrifuged (16,000 × g for 30 min) to generate particulate-free lysates. For immunoprecipitation experiments, proteins from whole cell lysates were incubated with control IgG, anti-V5 or HA antibody, each 5 μg, overnight at 4 °C before addition of 20 μl Protein G Sepharose for 1 h. Pellets were washed 3 times with PBS containing 0.3% CHAPS before solubilization of bound proteins in SDS sample buffer for 5 min at 95 °C, separation by SDS-PAGE, and detection of bound proteins by Western blot analysis. For nuclear lysates, HEK293T cells were incubated with nuclear lysis buffer (10 mM HEPES pH 7.9, 10 mM NaCl, 3 mM MgCl_2_, 1 mM DTT, 1 mM PMSF, aprotinin, 1 μg/ml, leupeptin, 1 μg/ml, 100 μM Sodium orthovanadate), nuclei were extracted by homogenization (Dounce Pestle B, 8 strokes), pelleted by centrifugation (800 × g for 3 min at 4 °C), and washed twice with nuclear lysis buffer (pellet 1). Supernatants were centrifuged at 100,000 × g for 1 h at 4 °C to generate the cytosolic (supernatant) and membrane (pellet 2) fractions. The pellets were washed twice with nuclear lysis buffer before sonication (3–4 short bursts on medium setting). Proteins from the nuclear (pellet 1), cytosolic (supernatant), and membrane fractions (pellet 2) were separated by SDS-PAGE and the indicated proteins were detected by Western blot analysis. Purity of nuclear, cytosolic, and membrane fractions were confirmed by detection of α-acetylated histone H3, enolase, and calnexin antibody by Western blot.

### Fluorescence imaging of STAT1 and PKCδ

For detection of endogenous PKCδ, COS7 cells were fixed, permeabilized, incubated with αPKCδ antibody (Santa Cruz) followed by Alexa 488 secondary antibody, and mounted in DAPI-containing solution as previously described for endogenous STAT1^12^. For detection of recombinant CFP-STAT1 or GFP-PKCδ localization, COS7 cells were transfected with plasmids before exposure to rapamycin and fixation with 4% paraformaldehyde for 15 min at room temperature. Construction of pcDNA3.1/nV5/ECFP-DEST-STAT1 was previously described^13^. The same approach was used to construct pcDNA3.1/nV5/GFP-PKCδ WT and PKCδ ΔTOS. After heterologous expression in COS7 cells, fluorescence was detected by multi-track image acquisition (ECFP: Excitation 458 nm, Emission 475 nm; GFP: Excitation 488 nm, Emission 594 nm; DAPI: Excitation 405 nm, Emission 475 nm) using a Plan-Neofluar 40 × /1.3x oil DIC objective and Zeiss LSM 510 META scanning confocal microscope. Images were acquired as previously described^12^. For endogenous PKCδ, nuclei were selected before measurement of pixel intensity using Image J, and results are expressed as mean nuclear density in 7–8 fields low powered fields per condition.

### Protein fragment complementation assay

Gateway destination plasmid vectors for protein fragment complementation assays (PCA) was described previously^13^. cDNAs encoding wild-type raptor, wild-type PKCδ, PKCδ TOS mutants, or KPNA1 were cloned by recombination into pcDNA3.1-based PCA mammalian expression vectors 3′ to each of the complementary fragments of *venus* yellow fluorescence protein (VF1, VF2). Vector plasmids, each 1 μg/9.6 cm^2^ dish surface area, were co-transfected in a 1:1 ratio (*e.g*., VF1-raptor:VF2-PKCδ) by liposomal transfection (Lipofectamine LTX Reagent, Invitrogen). For each biological replicate, cells were counted, and fluorescence was measured (Excitation 512 nm, Emission 529 nm) using a SpectraMax M2 Fluorometer (Molecular Devices). Expression was also assessed by Western blot. Data are expressed as fluorescence units per cell.

### *In vitro* kinase assay

Purification of GST-KPNA1 and GST-S6 from crude bacterial lysates was performed by immobilization on glutathione-Sepharose (GE Healthcare), before recovery in elution buffer (50 mM Tris-HCl, pH 8, 40 mM glutathione), and verification by Coomassie blue staining and Western blot analysis. Purified active PKCδ was obtained from Upstate. Kinase assays were performed for 10 min at 30 °C with recombinant GST-KPNA1 or GST-S6 (each 2 μg per assay), and 40 μM ATP with γ[^32^P]-ATP, 5 μCi, in kinase buffer (25 mM HEPES pH 7.4, 50 mM NaCl, 50 mM β-glycerophosphate, 10 mM MgCl_2_). All samples were subjected to SDS-PAGE and incorporation of radioactive phosphate [^32^P] was imaged using a Fuji PhosphorImager.

### Measurement of apoptosis by flow cytometry

COS7 cells were transfected with either pDEST53-PKCδ or pDEST53-PKCδ-IVMEF, and after 48 h cells were exposed to vehicle, 50 nM rapamycin, or 250 U/ml IFN-β for the indicated times before incubation with a PE-AnnexinV and 7-amino-actinomycin D (7-AAD) Apoptosis Detection Kit I (BD Biosciences) as per manufacturer’s instructions. Flow cytometry was performed using BD Fortessa X-20 (BD Biosciences) with FACSDiva Software (BD Biosciences). Subsequent analysis was performed using FlowJo Software Version 10.2 (Tree Star).

## Supplementary information

Supplementary Information
