# Publisher Correction: Regulation of protein kinase Cδ Nuclear Import and Apoptosis by Mechanistic Target of Rapamycin Complex-1

**DOI:** 10.1038/s41598-020-58962-z

**Published:** 2020-02-19

**Authors:** Antonio Layoun, Alexander A. Goldberg, Ayesha Baig, Mikaela Eng, Ortal Attias, Kristoff Nelson, Alexandra Carella, Nahomi Amberber, Jill A. Fielhaber, Kwang-Bo Joung, T. Martin Schmeing, Yingshan Han, Jeffrey Downey, Maziar Divangahi, Philippe P. Roux, Arnold S. Kristof

**Affiliations:** 1 Meakins-Christie Laboratories and Translational Research in Respiratory Diseases Program, Research Institute of the McGill University Health Centre, Faculty of Medicine, Departments of Medicine and Critical Care, 1001 Décarie Boulevard, EM3.2219, Montreal, Québec H4A 3J1 Canada; 2 Department of Biochemistry, McGill University, Montréal, Québec H3G 0B1 Canada; 3 Meakins-Christie Laboratories and Translational Research in Respiratory Diseases Program, Research Institute of the McGill University Health Centre, 1001 Décarie Boulevard, EM3.2219, Montréal, Québec H4A 3J1 Canada; 4 Institute for Research in Immunology and Cancer, Faculty of Medicine, University of Montreal, P.O. Box 6128, Station Centre-Ville, Montréal, Québec H3C 2J7 Canada

Correction to: *Scientific Reports* 10.1038/s41598-019-53909-5, published online 26 November 2019

In the original version of this Article, the author T. Martin Schmeing was incorrectly indexed. This error has now been corrected.

