# Supplementary material for: Regulation of protein kinase Cδ Nuclear Import and Apoptosis by Mechanistic Target of Rapamycin Complex-1
Source: Sci Rep. 2019 Nov 26;9:17620. doi: 10.1038/s41598-019-53909-5 (PMC6879585; doi:10.1038/s41598-019-53909-5)

Supplemental Materials

Regulation of protein kinase Cδ Nuclear Import and Apoptosis by Mechanistic Target of Rapamycin Complex 1

Antonio Layoun, Alexander A. Goldberg, Ayesha Baig, Mikaela Eng, Ortal Attias, Kristoff Nelson, Alexandra Carella, Nahomi Amberber, Jill A. Fielhaber, Kwang-Bo Joung, T. Martin Schmeing, Yingshan Han, Jeffrey Downey, Maziar Divangahi, Philippe P. Roux, and Arnold S. Kristof

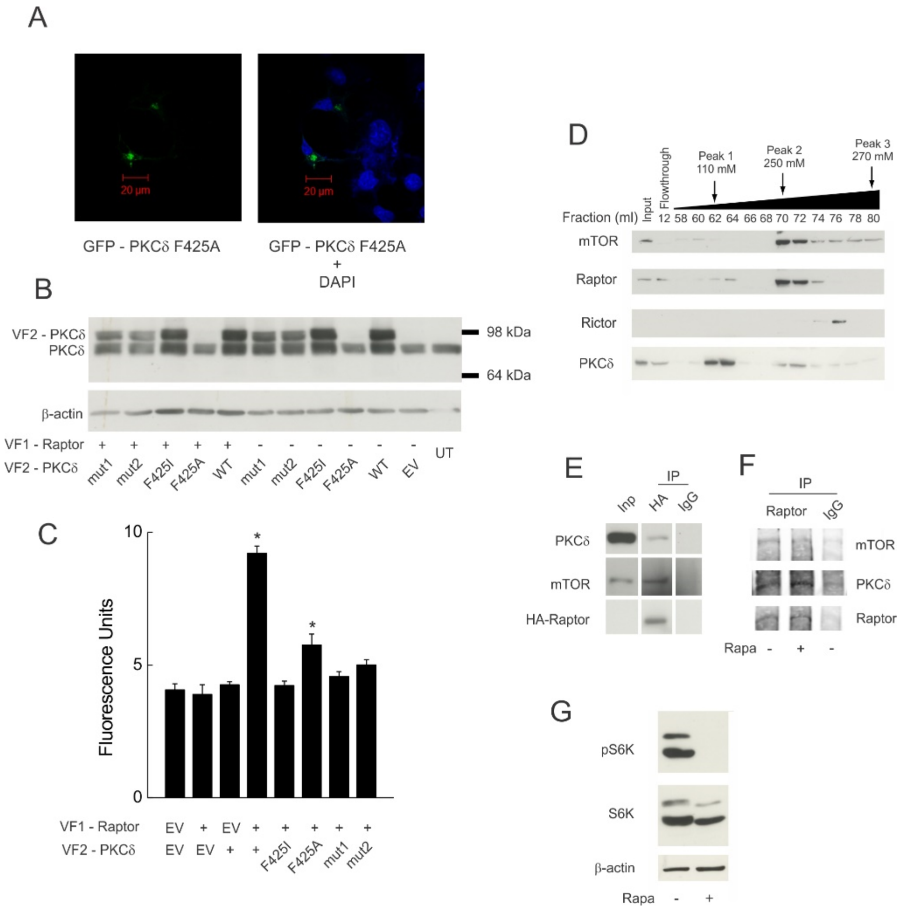

Fig. S1: A. Confocal images of COS7 cells expressing the GFP-V5-PKCδ F425A mutant (green) and DAPI-stained nuclei (blue). **B.** Western blot with α-PKCδ antibody in lysates from COS7 cells expressing full-length recombinant wild-type VF2-PKCδ (WT) or that with its TOS motif (5'-FVMEF-3') mutated to the homologous sequence found in STAT3 (5'-FPMEL-3', mut1) or S6K (5'-FDLDL-3', mut2) (see Fig. 1A). Also shown are detection of the F425A and F425I TOS mutants. Endogenous PKCδ and β-actin are indicated below VF2-PKCδ. **C.** Recombinant VF2-PKCδ interactions with VF1-raptor by protein fragment complementation assay (PCA) in COS7 cells transfected with empty vector (EV) or that for expression of VF2-PKCδ mut1, mut2, F425A, or F425I. **D.** HEK293T whole cell homogenates were applied to a SP Sepharose column (GE Healthcare) and bound proteins were eluted by continuous NaCl gradient, and collection in 1-ml fractions. Proteins in fractions from chromatographic peaks were detected by Western blot analysis. **E.** After expression of HA-raptor in HEK293T cells, PKCδ, HA-raptor, and mTOR were detected by Western blot in complexes affinity purified using αHA antibody. Composite images from the same gel are shown, with lanes separated by vertical white lines. **F.** In HEK 293T cells exposed to vehicle or 50 nM rapamycin (rapa), mTOR, PKCδ, and raptor were affinity purified using antibody recognizing endogenous raptor. **G.** Whole cell lysates used for immunoprecipitation in Fig. 2A were probed for phosphor-p70 S6 kinase (T389), total p70 S6 kinase, and b-actin by Western blot analysis.

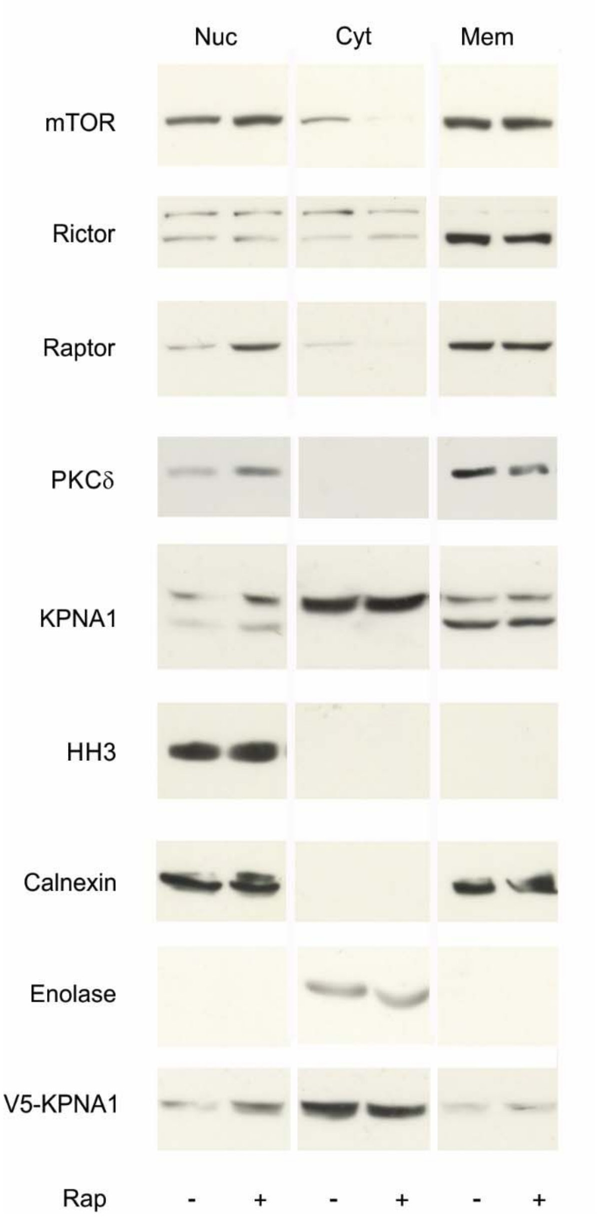

Fig. S2: Nuclear enrichment of proteins. Fractionated lysates from cells exposed to vehicle or rapamycin (Fig. 2C) were prepared as indicated in the Methods section. The indicated proteins were detected in nuclear, cytosolic, and membrane fractions by Western blot analysis. Nuclear and cytosolic fractions are indicated by expression of acetylated histone H3 (HH3) and enolase, respectively. Calnexin is present in the membrane fraction. Note detection of calnexin in the nuclear lysates due to endoplasmic reticulum contiguous with the nuclear membrane.

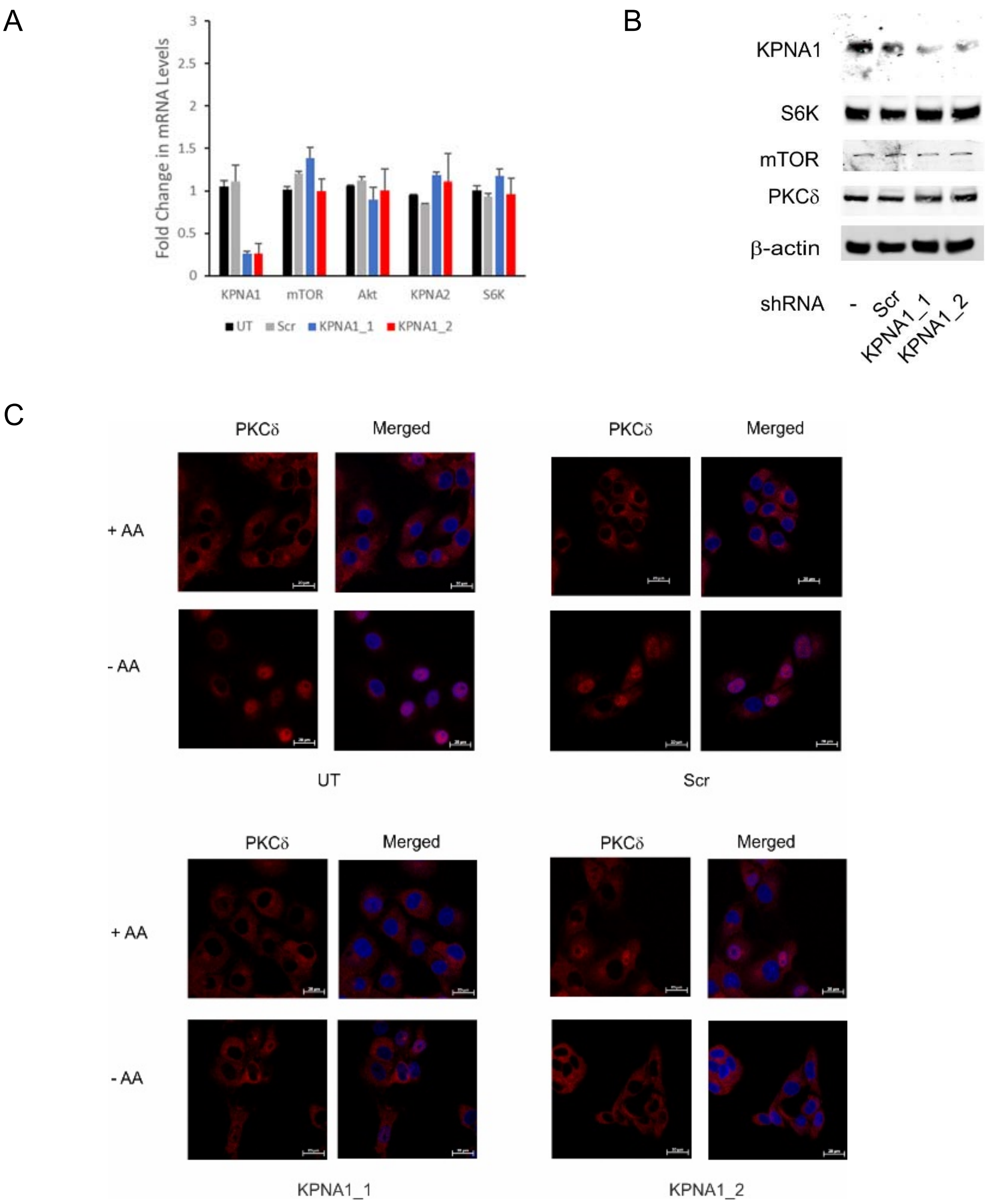

Fig. S3: KPNA1 Target Validation and Biological Relevance: Untransduced (UT, black) COS7 cells or those expressing scrambled control (Scr, grey) or KPNA1-targeting shRNAs (blue and red) were exposed to **A**. and **B.** growth media before evaluation of levels of the indicated mRNAs by qPCR (primer sequences listed in Table S4), or **C.** complete RPMI medium (+AA) or RPMI lacking leucine, arginine, and lysine (-AA) for 1 h before confocal imaging of endogenous PKCδ (red) or nuclei (blue). In A. Data are mean fold-change *vs.* control = 1 of triplicate samples from 2 experiments (± half the range) as determined by the ΔΔCT method. In B., COS7 cells were cultured and homogenized before measurement of the indicated proteins by Western blot analysis.

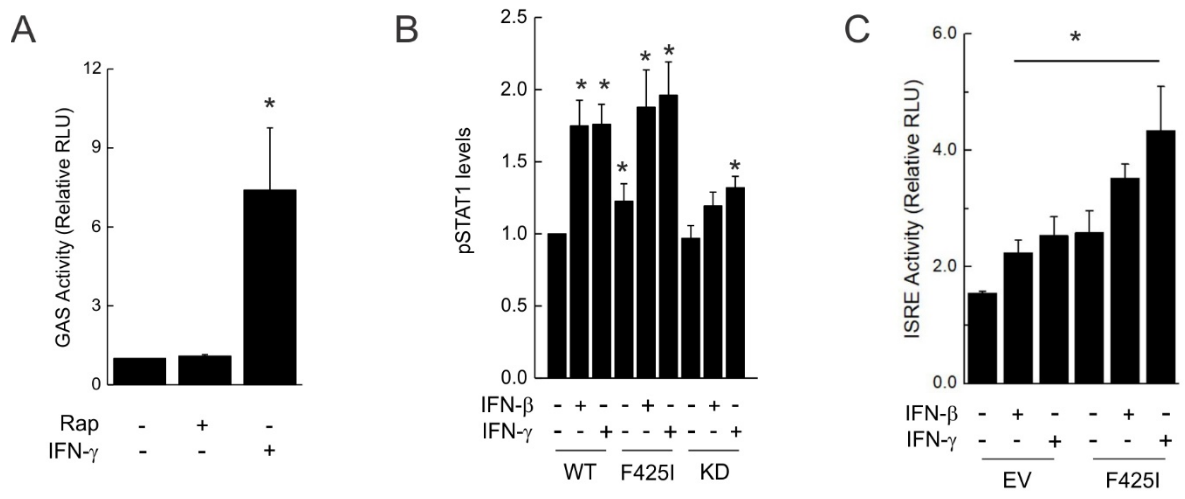

Fig. S4: Effect of TOS mutation or rapamycin on STAT1 transcriptional activity. **A.** A549 cells were transfected with pGAS-TA-luc (Clontech) before exposure to vehicle, 50 nM rapamycin, or IFN-γ, 200 U/ml for 6h and measurement of luciferase activity by luminometry (Dual Luciferase kit, Promega). **B.** HEK293T cells expressing wild-type (WT), dominant-negative (DN) or TOS-mutated (F425I) PKCδ were exposed to IFN-γ, 200 U/ml, or IFN-β, 250 U/ml, for 30 min before measurement of phospho-STAT1 S272 (pSTAT1) by Western blot in whole cell lysates. Densitometric quantification of pSTAT1 levels is shown in in Panel B (means of fold changes (± SEM) in pSTAT1 levels from 3 individual experiments. * $p < 0.05$ *vs.* vehicle control in wild-type PKCδ-expressing cells. **C.** Cells were transfected with pISRE-TA-luc (Clontech) before exposure to vehicle, IFN-β (250 U/ml), or IFN-γ (200 U/ml) for 6h and measurement of luciferase activity by luminometry (Dual Luciferase kit, Promega). Shown are the means of normalized luciferase activity (± SEM) from 3 individual experiments * $p < 0.05$ *vs.* vehicle control in empty vector-transfected cells.

Supplementary Tables:

Table S1: Oligonucleotide primers (5'-3') for cloning of raptor and PKCδ:

Plasmid	Forward Primer	Reverse Primer	Template
pDONR 221 – PKCδ	GGGGACAAGTTTGTACAA AAAAGCAGGCTTCATGGC GCCGTTCCTGCGCATC	GGGGACCATTTGTACAAGA AAGCTGGGTTCTAACCGGAA CCTCCATCTTC	pcDNA-PKCδ
pDONR 221 – Raptor	GGGGACAAGTTTGTACAA AAAAGCAGGCTTCATGGA GTCCGAAATGCTGCAAT	GGGGACCACTTTGTACAAGA AAGCTGGGTCCTATCTGACA CGCTTCTCCACCGA	pRK5-HA-Raptor/pRK5

Table S2: Oligonucleotide primers (5'-3') for PKCδ site-directed mutagenesis with pDONR221-PKCδ as template:

Mutation	Forward Primer	Reverse Primer
PKCδ F425I (ΔTOS)	GGACCACCTGTTC ATT GTG ATGGAGTTC CTCAACGG	CCGTTGAG GAA CTC CATCAC AATGAACAGGTGGTCC
PKCδ F425A	GGACCACCTGTTCGCTGTG ATGGAGTTC CTCAACGG	CCGTTGAGGAACTCCATCAC AGCGAACAGGTGGTCC
PKCδ mut1	GGACCACCTGTTCTTTCCG ATGGAGCTCCTCAACGG	CCGTTGAGGAGCTCCATCGG AAAGAACAGGTGGTCC
PKCδ mut2	CCAGACCAAGGACCACCTGTTCTTTG ATTGGACCTCCTCAACGG	CCGTTGAGGAGGTCCAAGTC AAAGAACAGGTGGTCCTTGGTCTGG
PKCδ K378R (kinase dead)	GGAGAGTACTTTGCCATCAGGGCCCT CAAGAAGG	CCTTCTTGAGGGCCCTGATGGCAAAGT ACTCTCC

Table S3: Antibodies used for Western blot analysis or immunoprecipitations:

Protein/epitope	Source
Akt	Cell Signaling Technology
Cleaved Caspase-3	Cell Signaling Technology
HA	Millipore
Mouse anti-STAT1α (IP or Western)	Santa Cruz Biotechnology
mTOR	Millipore
phospho-P70 S6 kinase T389	Cell Signaling
phosphor-STAT1 S727	Cell Signaling
phospho-S6 S235/S236	Cell Signaling
PKCδ	Millipore
Rabbit anti-STAT1 (Western or IF)	Santa Cruz Biotechnology
Raptor	Cell Signaling Technology
Rictor	Cell Signaling Technology
p70 S6 kinase	Cell Signaling Technology
V5	Cell Signaling Technology
β-actin	Sigma

Table S4: Oligonucleotide primers (5’-3’) for Sybr Green-based Real-time PCR:

Transcript	Forward Primer	Reverse Primer
Akt	GAAGAGATGGAGGTGTCCCT	ATCTTCATGGCGTAGTAGCG
GAPDH	AAGAAGGTGGTGAAGCAGGCG	ACCAGGAAATGAGCTTGACAA
KPNA1	CAAATCTCTGAATCCCGATG	TTCTCCGCTTGAATAACTGC
KPNA2	ATACCTGCTGGGCTATTTCC	CTCTTAGGGCAGGAGTCACA
mTOR	GCCATCCAGATTGATACCTG	TGTCTGTGAGAAGCTGGTGA
S6K	AGCACAGCAAATCCTCAGAC	TCATTGTCACATCCATCTGC

Additional Supporting Materials

Original Images for Western blots in Figures 1, 2, 5, 6, S1-3

Legend:

☐ Box indicating the bands used for the Figure.

■ Box covering information or conditions that are not relevant to the manuscript.

Original blots Fig. 1C:

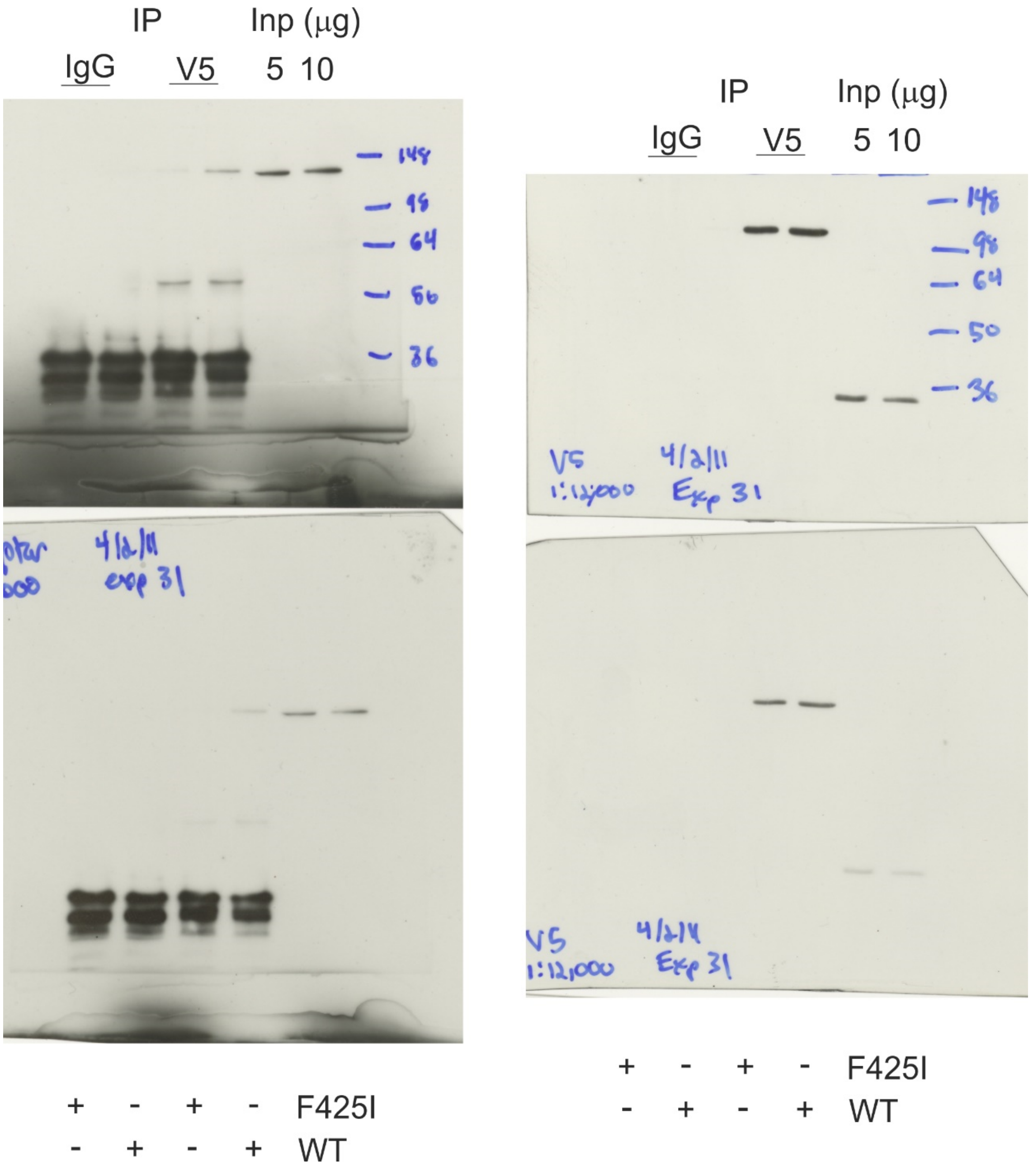

Original blots Fig. 1D:

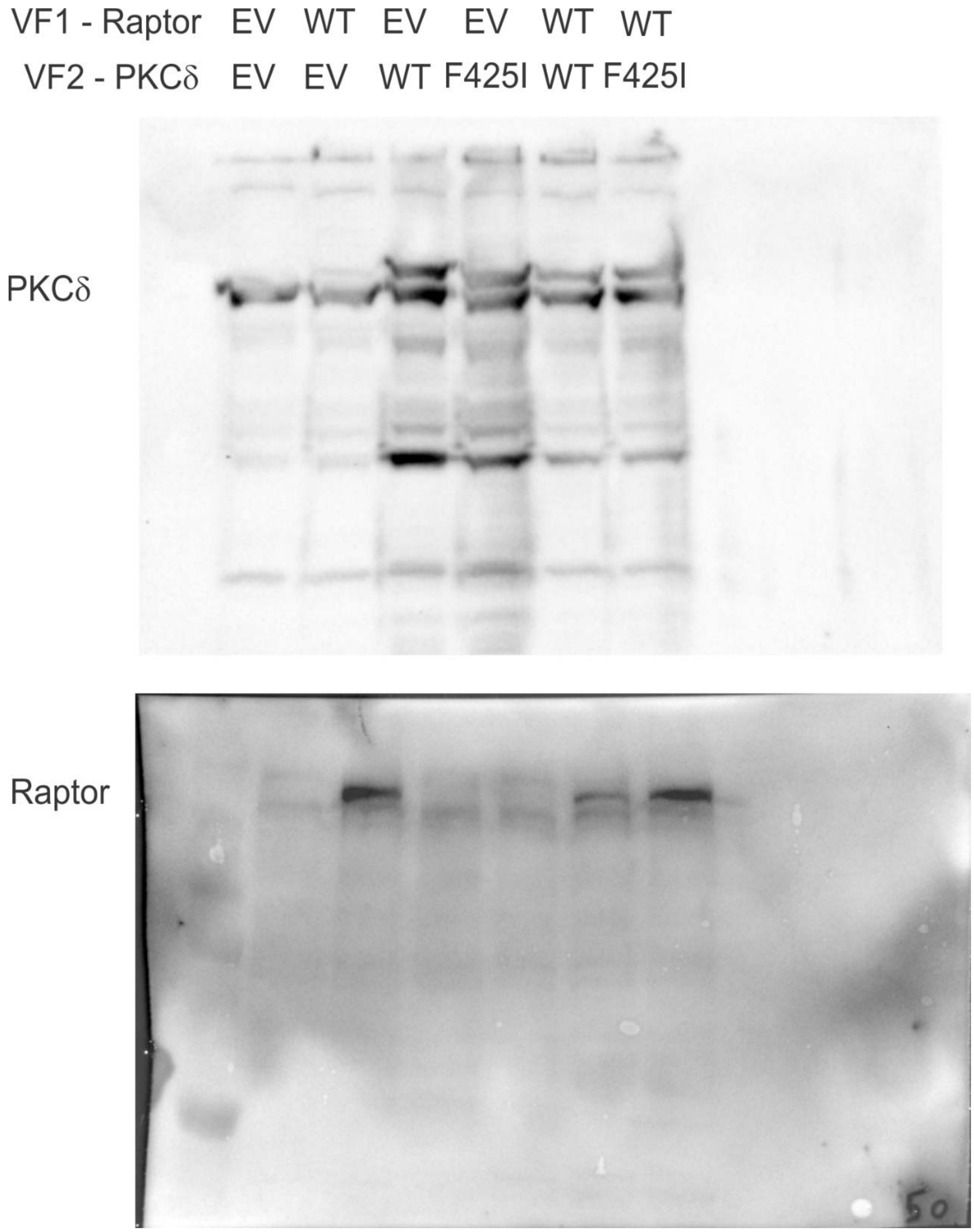

Original blots Fig. 2A:

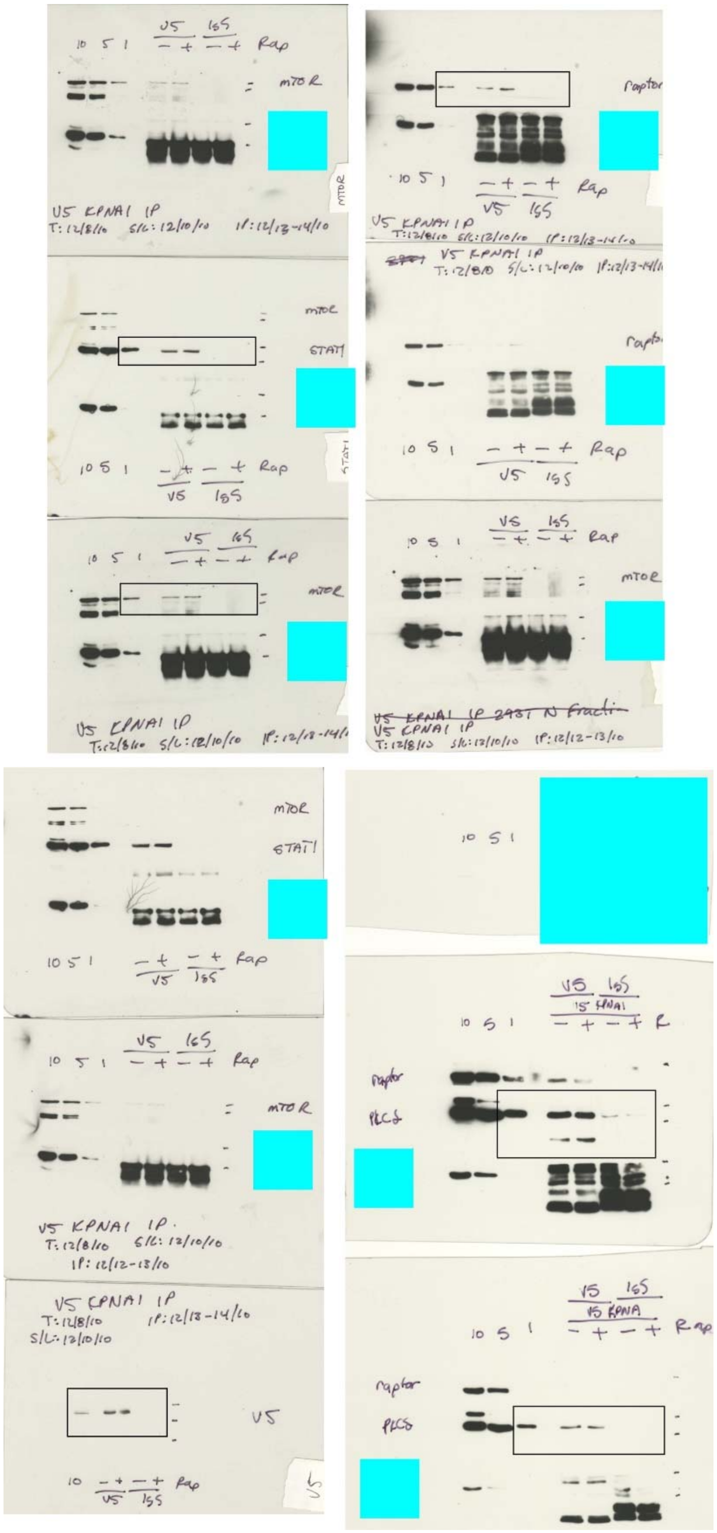

Original blots Fig. 2C:

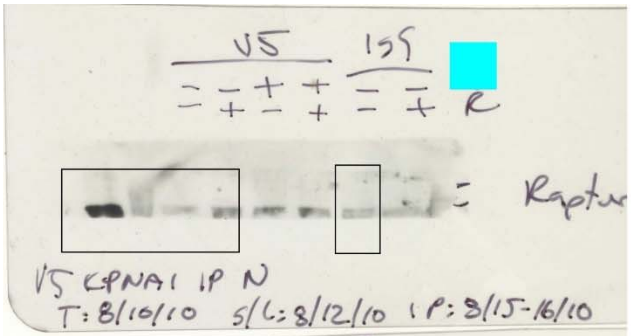

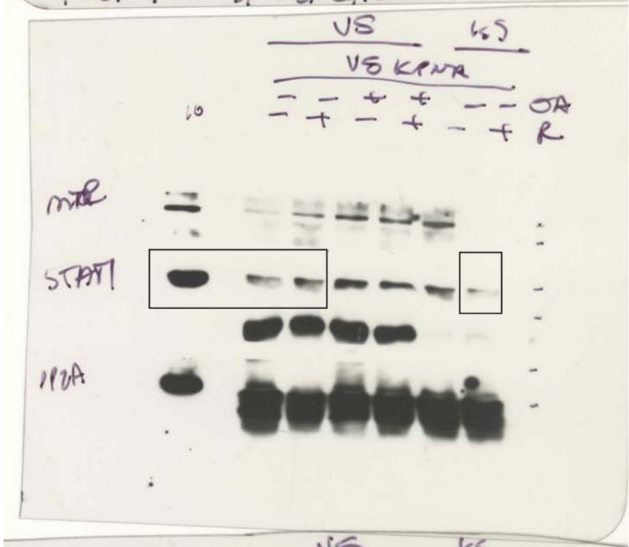

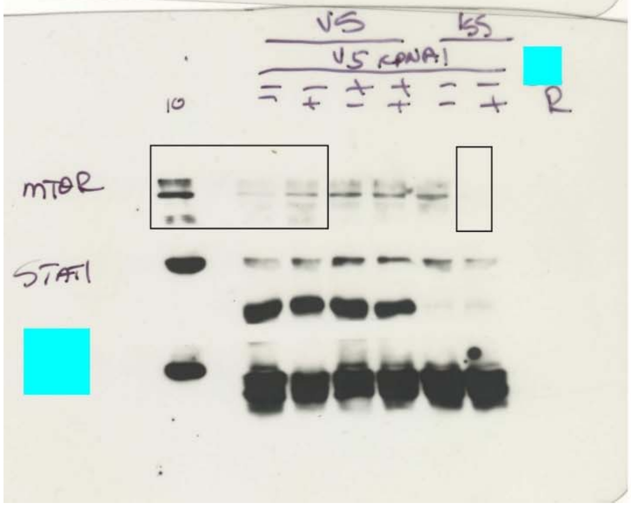

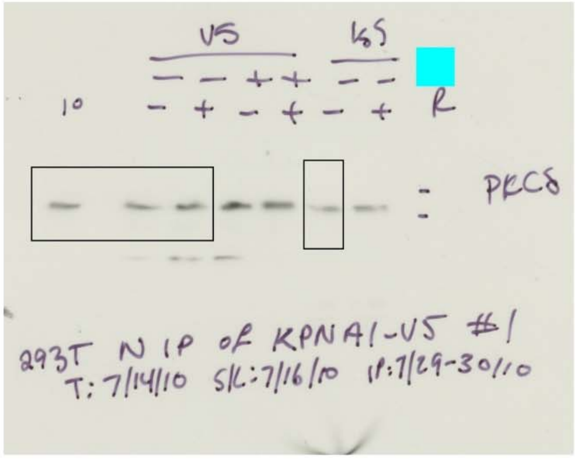

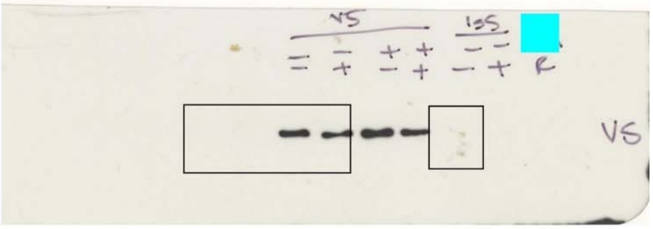

Original blots Fig. 6A:

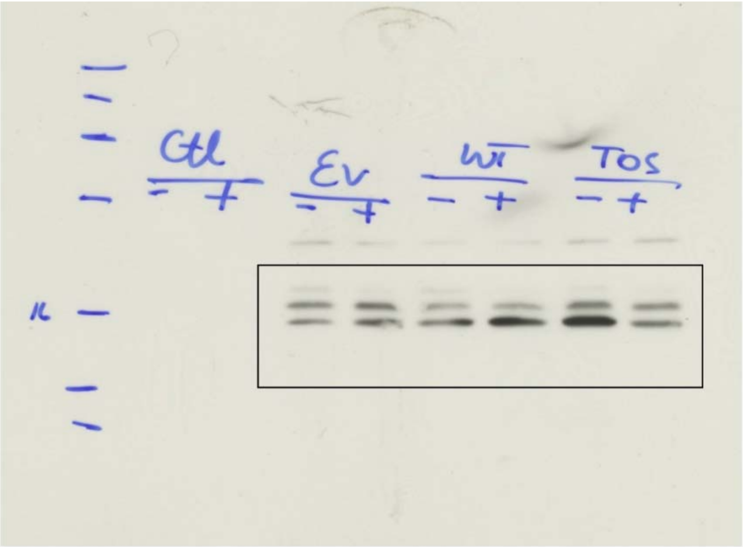

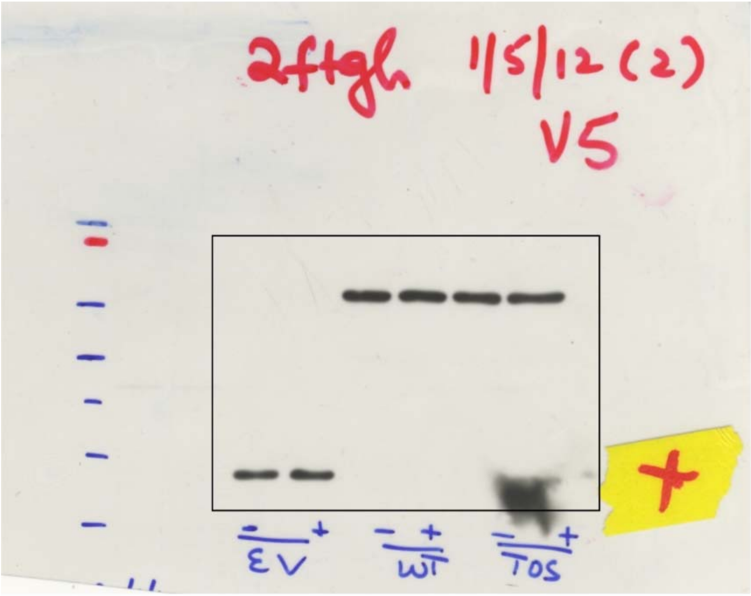

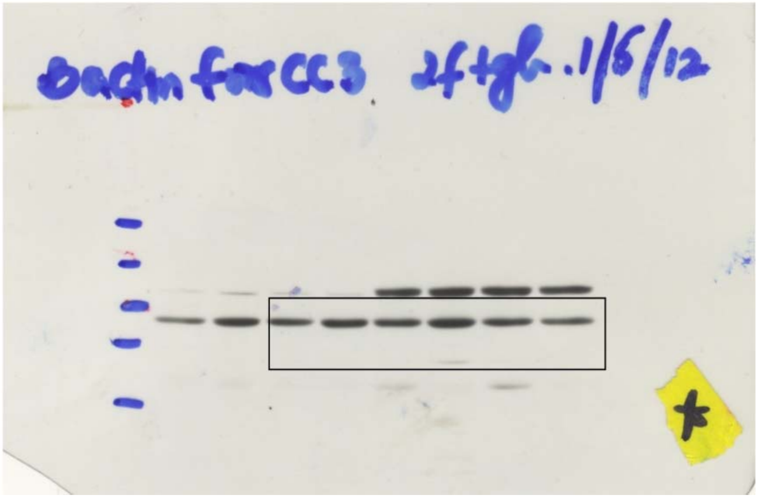

Original blots Fig. 6B:

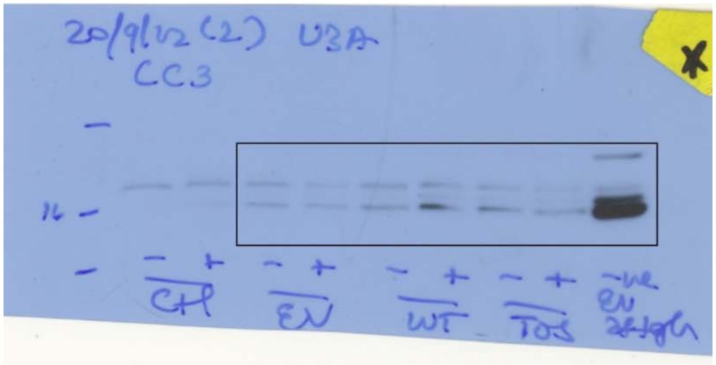

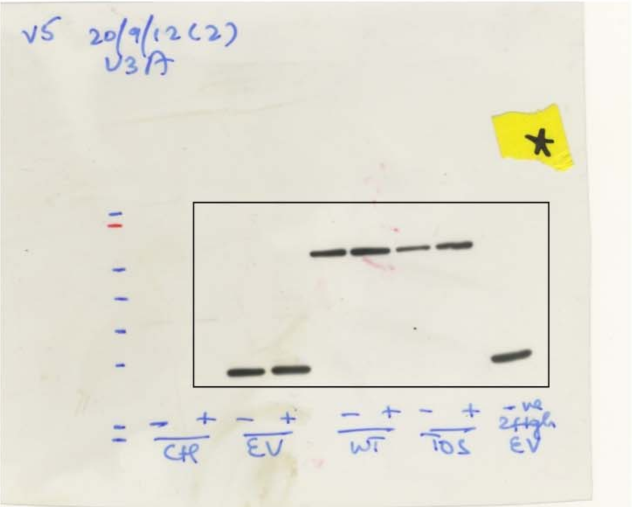

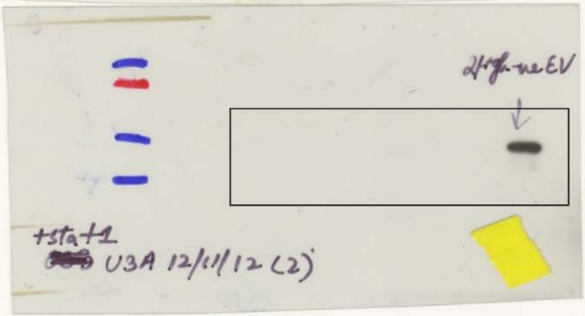

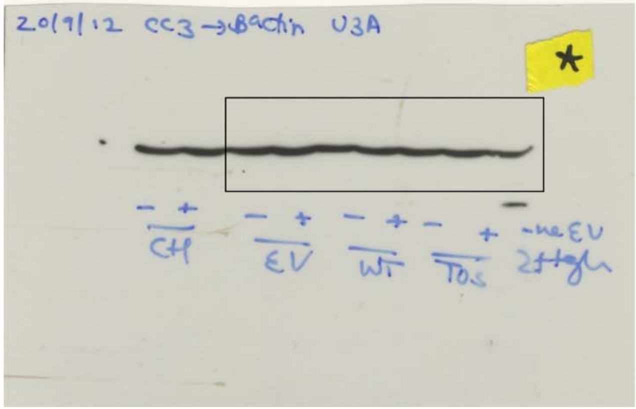

Original blots Fig. S1B and S1D:

B

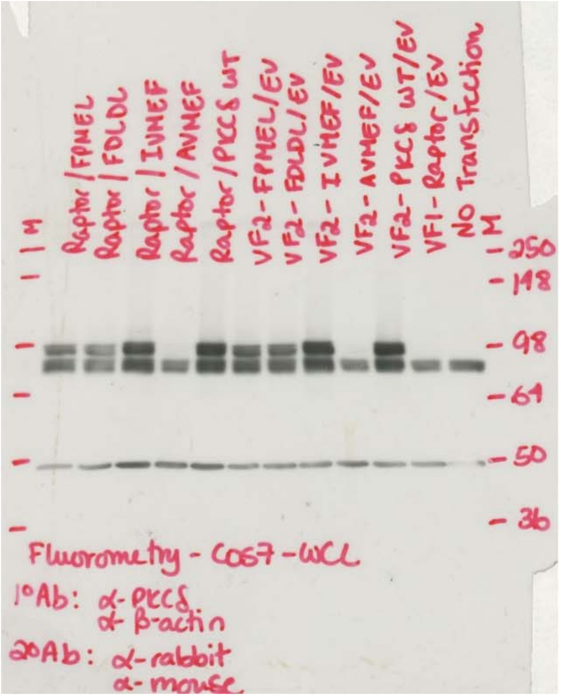

D

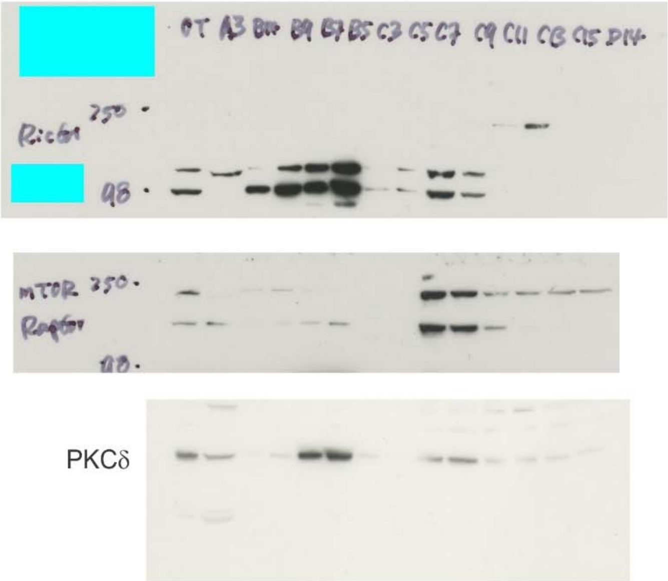

Original blots Fig. S1E:

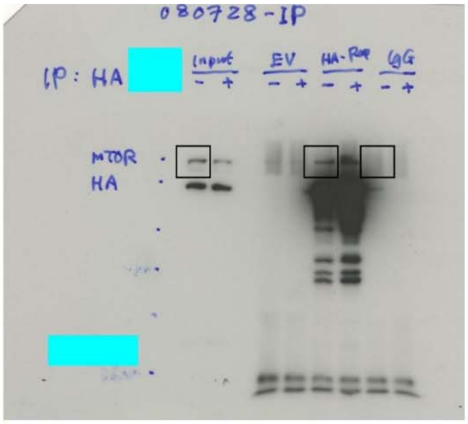

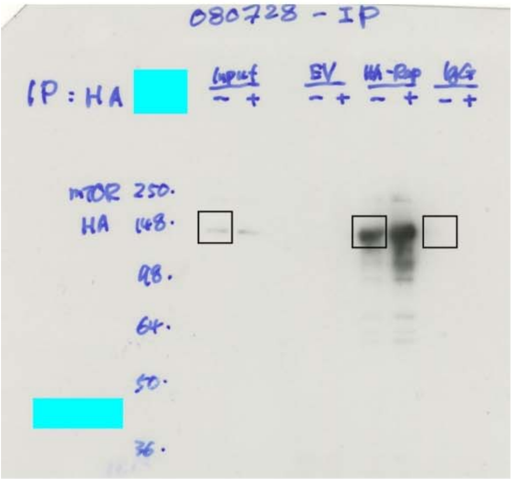

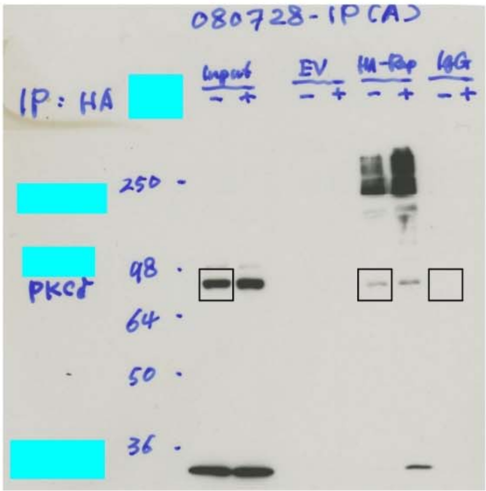

Original blots Fig. S1F, G:

Fig. S1F

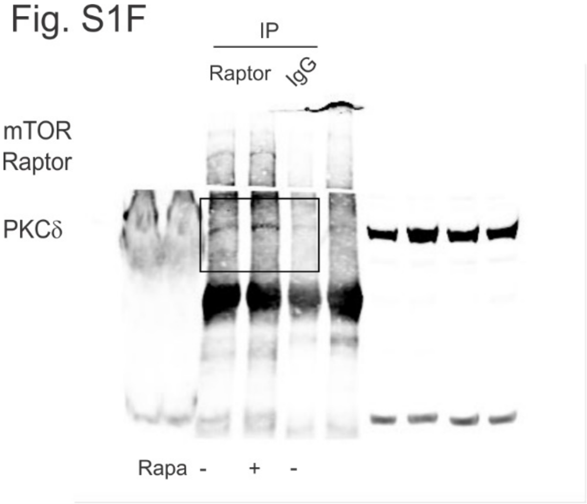

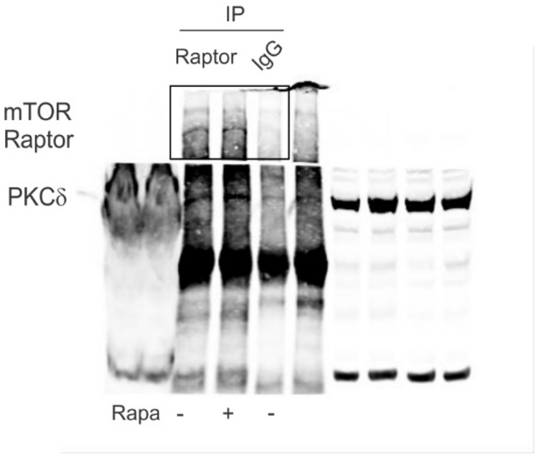

Fig. S1G

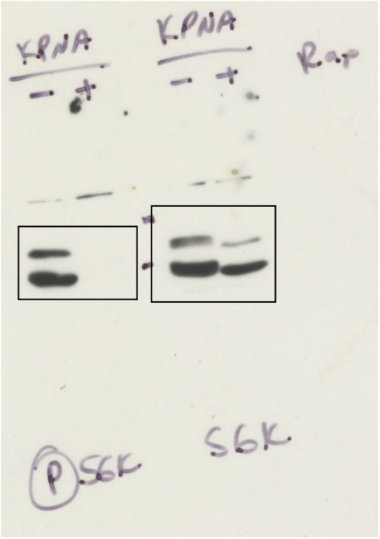

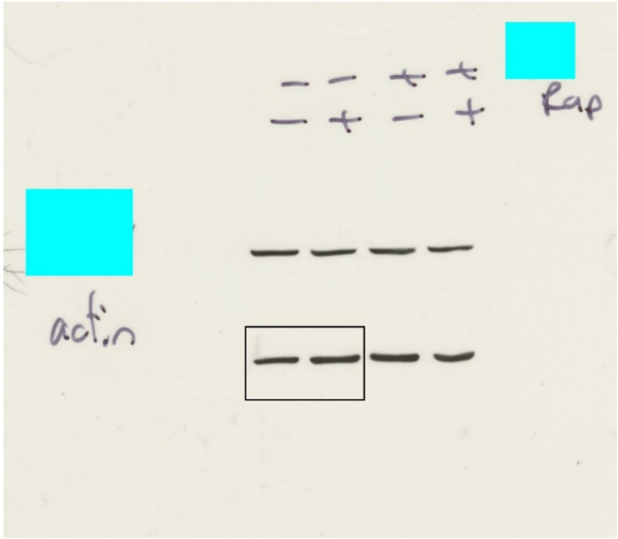

Original blots Fig. S2:

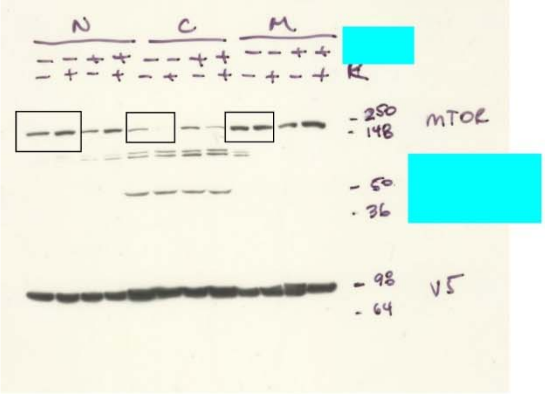

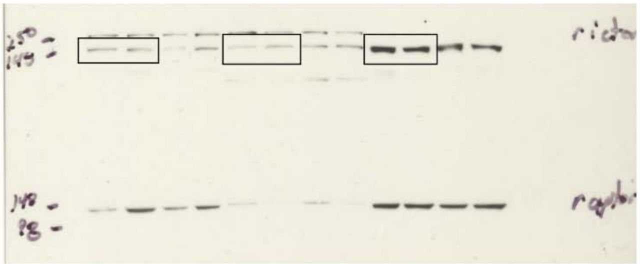

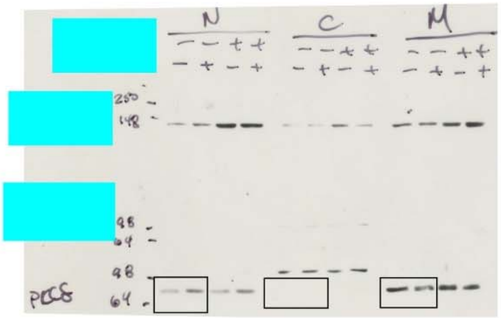

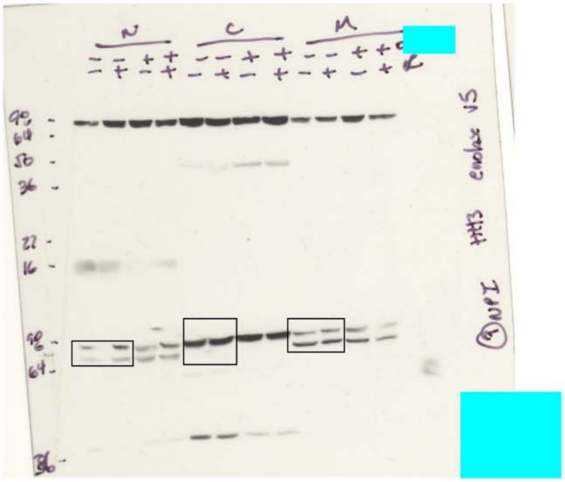

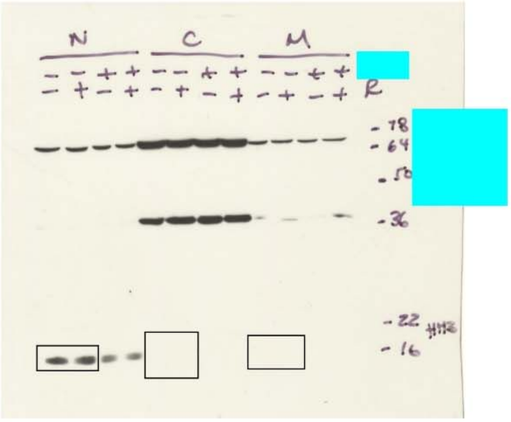

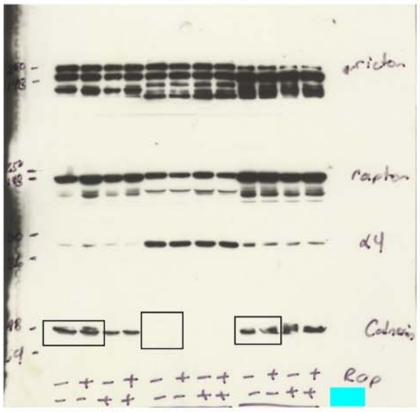

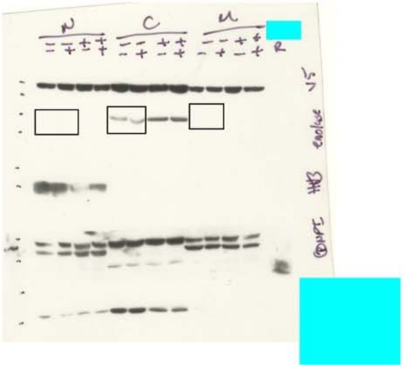

Original Blots (Lycor System) Fig. S3B:

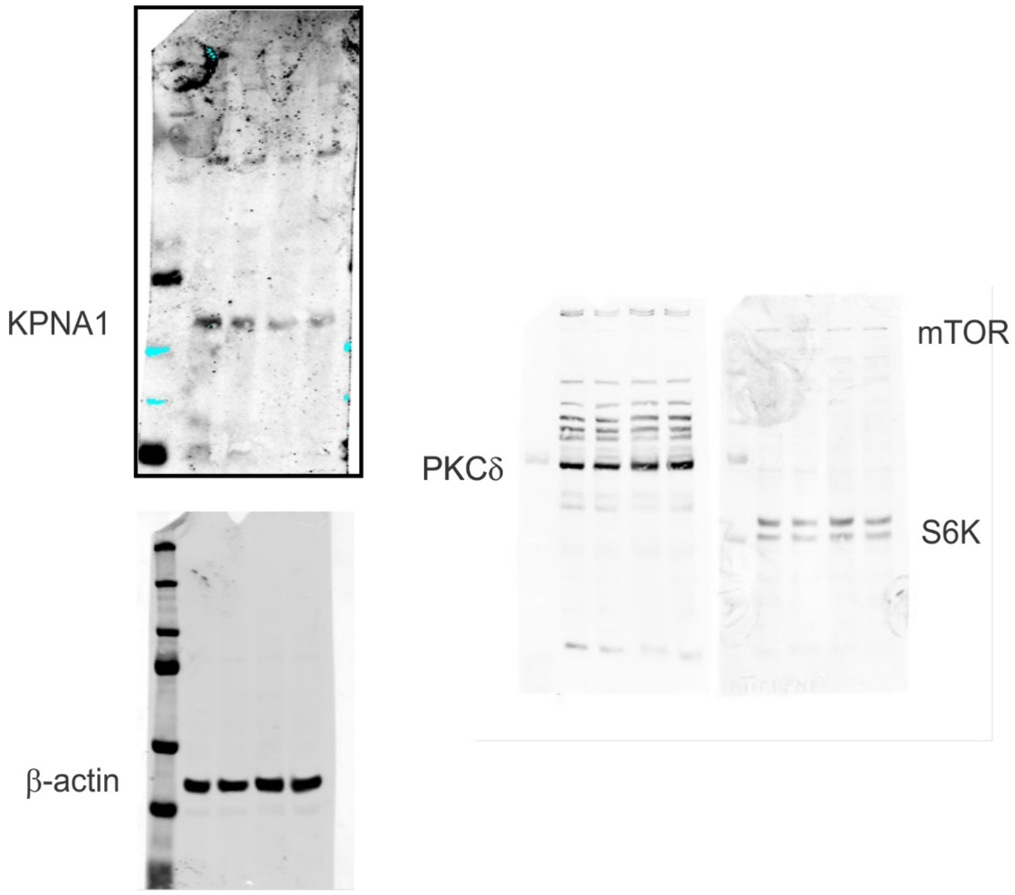

Supplement: Supplementary file 1 — Supplementary Information [file 41598_2019_53909_MOESM1_ESM.pdf]
